# Circular RNAs and their emerging roles in muscular immune-related diseases

**DOI:** 10.3389/fimmu.2025.1675567

**Published:** 2025-11-13

**Authors:** Felicita Urzi, Anja Srpčič, Katja Lakota

**Affiliations:** 1 Faculty of Mathematics, Natural Sciences and Information Technologies, University of Primorska, Koper, Slovenia; 2 Faculty of Medicine, Institute of pathophysiology, University of Ljubljana, Ljubljana, Slovenia; 3 Department of Rheumatology, University Medical Centre Ljubljana, Ljubljana, Slovenia

**Keywords:** circular RNA, skeletal muscle, immune cells, idiopathic inflammatory myopathies, Duchenne muscular dystrophy, myasthenia gravis

## Abstract

Circular RNAs (circRNAs) have recently emerged as a highly stable and versatile class of non-coding RNAs that play critical roles in gene regulation, yet their involvement in immune-mediated muscle disorders remains largely underexplored. This review synthesizes how circRNAs influence key processes in both skeletal muscle and immune cells, from myogenesis, regeneration, and muscle stem cell function to inflammatory signaling and muscle wasting. Our aim was to identify circRNA insights across muscle immune-mediated diseases. However, we found no idiopathic inflammatory myopathy-focused circRNA studies, only a limited body of work in Duchenne muscular dystrophy, and predominantly peripheral blood mononuclear cell-based evidence in myasthenia gravis. These gaps highlight clear priorities: subtype-resolved circRNA atlases for idiopathic inflammatory myopathy; paired muscle–biofluid and cell-type–resolved profiling (including infiltrating immune populations); rigorous *in vivo* functional validation beyond correlative expression; fuller mechanistic delineation beyond miRNA competition (e.g., RNA binding protein interactions, translation, epigenetic regulation); and longitudinal cohorts linking circRNA dynamics to disease activity and treatment response. We particularly noted lack of in-depth studies addressing the interplay between muscle and immune cells in these conditions. Furthermore, we examine pioneering efforts to engineer circRNAs as therapeutic agents, capable of either neutralizing pathogenic pathways that drive muscle atrophy or restoring dystrophin expression in genetic disease models. Finally, we outline future directions for circRNA profiling in patient tissues and biofluids, rigorous functional validation *in vivo*, and the development of circRNA-based diagnostics. This positions circRNAs at the forefront of next-generation strategies for understanding and combating immune-related muscular disorders.

## 1 Introduction

Muscular immune-related diseases encompassing conditions such as idiopathic inflammatory myopathies, Duchenne muscular dystrophy, and myasthenia gravis are characterized by persistent inflammation, immune dysregulation, and progressive muscle dysfunction (1–4). These diseases result in significant morbidity and diminished quality of life, yet their complex molecular underpinnings remain only partially understood.

The ongoing question concerns the underlying molecular mechanisms responsible for altered gene regulation and expression in these conditions, as well as the determinants of disease subtype and disease trajectory.

Recent advances in transcriptomic technologies have revealed the importance of non-coding RNAs in regulating immune responses and tissue integrity (5–7). Among these, circular RNAs (circRNAs) have emerged as a novel and functionally diverse class of regulatory molecules with unique properties that hold substantial relevance to immune-mediated muscle diseases (8–10).

CircRNAs are single-stranded RNA loop molecules with enhanced stability and resistance to exonuclease degradation (11). These characteristics, along with their tissue-specific expression patterns and evolutionary conservation (12), make circRNAs attractive candidates for both mechanistic study and clinical biomarker development. Initially considered transcriptional noise, circRNAs are now recognized as critical modulators of gene expression and cell signalling (13).

In skeletal muscle and immune cells, circRNAs are dynamically regulated during development, regeneration, and inflammation (8, 14). Evidence increasingly suggests that circRNAs participate in key signalling pathways involved in immune activation, muscle atrophy, and repair processes, such as Nuclear factor kappaB (NF-κB) (15, 16), Janus kinase (JAK)/signal transducer and activator of transcription (STAT) (17), Myostatin/Smad (18), and the cyclic GMP–AMP synthase (cGAS)–stimulator of interferon genes (STING) (19). CircRNAs exert multifaceted regulatory roles by influencing both processes intrinsic to muscle cells themselves and the behavior of extrinsic immune cells that interact with the muscle tissue. Intrinsic effects of circRNAs that can directly modulate the inherent functions and responses within muscle cells include regulating their capacity for myogenesis, regeneration (repair after injury), survival, metabolic activity, or their susceptibility to damage and atrophy. Significant evidence for these roles comes from studies on normal muscle development (20), regeneration models (21), and conditions like Duchenne muscular dystrophy where muscle cell intrinsic defects are a primary cause (22). Extrinsic effects pertain to how circRNAs govern the activities of immune cells that impact muscle tissue. This involves influencing immune cell recruitment and infiltration into the muscle, their activation and polarization (e.g., towards pro-inflammatory M1 or anti-inflammatory M2 macrophage phenotypes (23); Th1, Th2, or Th17 T-cell differentiation), and their production and release of signalling molecules like cytokines and chemokines (24). Insights into these roles are often derived from general immunology studies, models of muscle injury with inflammatory components, and diseases like myasthenia gravis or Duchenne muscular dystrophy where immune responses are significantly involved (22, 25).

Their diverse regulatory potential underscores the possible role of circRNAs in the pathology of muscular immune-related diseases. This review sought new insight into muscle immune-mediated diseases through the lens of circRNAs; in so doing, we identified a substantial gap—no idiopathic inflammatory myopathy-specific circRNA studies, limited evidence in Duchenne muscular dystrophy, and myasthenia gravis data largely confined to peripheral blood mononuclear cells (PBMCs). As circRNA research advances, these molecules hold substantial promise for redefining the molecular mechanisms and treatment strategies of immune-mediated muscular disorders.

## 2 Biogenesis and functions of circRNAs

CircRNAs represent a distinct class of endogenous RNA molecules characterized by their unique covalently closed loop structure, formed through a process known as back-splicing (26). This process involves the joining of a downstream splice donor site to an upstream splice acceptor site, resulting in a circular transcript that lacks the 5’ and 3’ ends typical of linear RNA molecules. This structural feature confers upon circRNAs a remarkable stability, rendering them resistant to degradation by RNA exonucleases, which typically target linear RNAs. The average half-life of circRNAs can be significantly longer than that of linear mRNAs, often exceeding 48 hours. This inherent stability positions circRNAs as potentially long-lasting regulators of cellular processes and promising candidates for therapeutic applications and biomarkers (27, 28).

CircRNA biogenesis via back-splicing occurs co-transcriptionally using the canonical spliceosome machinery. The formation of a circRNA is often in direct competition with the canonical splicing of its linear mRNA counterpart from the same pre-mRNA transcript (29). Several factors influence whether a pre-mRNA molecule will undergo back-splicing to form a circRNA or canonical splicing to yield a linear mRNA. This balance is tightly regulated and can be influenced by:

i) The strength of canonical splice sites: i.e. weak canonical 5’ and 3’ splice sites around an exon or group of exons can favor back-splicing;
ii) The presence and length of intronic complementary sequences: i.e. Alu repeats within long flanking introns can base-pair, bring splice sites into proximity and facilitate the nucleophilic attack required for back-splicing;
iii) Cis-acting sequence motifs: Specific motifs within the introns or exons can act as enhancers or silencers of either canonical splicing or back-splicing; i.e. the Quaking Response Element (QRE), a specific cis-acting sequence motif with the core sequence ACUAAC, can act as a powerful enhancer of back-splicing.
iv) RNA-binding proteins (RBPs): These trans-acting factors can bind to flanking intronic regions, acting as enhancers or repressors of circularization, i.e. QKI (Quaking) binds to Quaking Response Elements in flanking introns and dimerizes, bringing splice sites together to promote circularization, while ADAR1 (Adenosine Deaminase Acting on RNA 1) can inhibit back-splicing by editing and destabilizing the dsRNA structures formed by intronic complementary sequences.

These cis-regulatory elements and trans-acting factors are critical for the specific expression patterns of circRNAs within muscle and other tissues (28, 30, 31).

Recent research shows that circRNAs could be regulated through epigenetic modifications such as methylation (32). The N^6^-Methyladenosine (m^6^A) modification on circRNAs can significantly influence their back-splicing efficiency during biogenesis and their cytoplasmic export. For example, m^6^A -modified circNSUN2 is exported from the nucleus to the cytoplasm in an m^6^A-dependent manner (32). m^6^A modification on circRNAs also affect stability, translation and degradation of circRNA (33).

Based on their genomic origin and composition, circRNAs are primarily classified into (34, 35):

i) Exonic circRNAs (ecircRNAs): The most common type, composed solely of one or more back-spliced exons. They are predominantly cytoplasmic and function as microRNAs (miRNA) sponges or protein interactors;
ii) Intronic circRNAs (ciRNAs): Formed entirely from introns that escape debranching. They are typically nuclear and can regulate parental gene transcription;
iii) Exon-Intron circRNAs (EIciRNAs): Contain both exons and retained introns. Like ciRNAs, they are mainly nuclear and can modulate parental gene transcription and splicing.
Beyond these, other less common types contribute to circRNA diversity, including antisense circRNAs (transcribed from antisense strands) and intergenic circRNAs (originating from intergenic regions), whose functions are generally less understood (35).
Recent discoveries have further expanded this classification to include unique exon-derived types:
i) Fusion circRNAs (f-circRNAs): Result from genomic rearrangements (i.e., translocations) joining exons from different genes into a single circular molecule, often associated with cancer (36);
ii) Readthrough circRNAs (rt-circRNAs): Generated from transcriptional readthrough events where transcription proceeds into an adjacent gene, followed by back-splicing involving exons from both loci, typically expressed at low levels (37, 38).

The significance of circRNAs in gene regulation is increasingly being recognized (13). They exert their regulatory functions through diverse mechanisms, such as binding to other RNA molecules or translating into proteins. One of the best-known functions of circRNAs is their ability to act as competitive endogenous RNAs (ceRNAs) by sponging miRNAs and preventing them from binding target mRNAs (39, 40). Although the miRNA sponge model is a classic mechanism, recent studies indicate that only a subset of circRNAs exhibit significant miRNA sponge activity. Quantitative analyses suggest that effective ceRNA competition is limited to highly abundant circRNAs with specific binding-site architectures, thus restricting the generality of the sponge model *in vivo*. This binding is typically investigated using dual-luciferase reporter assays, where the circRNA’s ability to nullify the suppressive effect of a miRNA on a downstream reporter gene is measured. Crucially, control experiments often demonstrate that the linear counterpart of the circRNA does not exhibit the same potent miRNA-sequestering effect, thus highlighting the structural importance of the circular form for this interaction. Moreover, results from luciferase reporter assays confirming circRNA-miRNA interactions should be interpreted cautiously without robust dose-response and stoichiometric analyses (41). In addition, circRNAs may influence miRNA storage, sorting, and localization, expanding their regulatory roles (42). Moreover, circRNAs contribute to RNA-protein interactions by serving as protein sponges, scaffolds, and modulators (43) or interacting with RBPs (44).

Beyond structural and trafficking roles, m^6^A modifications can even enable cap-independent translation of certain circRNAs, thereby expanding their functional repertoire to include protein-coding potential. Furthermore, m^6^A modifications can empower circRNAs to exert downstream effects, such as stabilizing target mRNAs (like HMGA2 in colorectal cancer) through complexes formed with m^6^A reader proteins (45).

Certain circRNAs, particularly ciRNAs and EIciRNAs, are enriched in the nucleus and modulate transcriptional processes. ciRNAs, such as ci-ankrd52, accumulate at transcription sites and enhance RNA polymerase II (Pol II) activity, while ci-sirt7 interacts with the elongating Pol II complex to promote transcription of the parental SIRT7 gene. Interfering with these circRNA function reduced the expression of parental genes, indicating their regulatory role in Pol II elongation (34). Similarly, EIciRNAs circEIF3J and circPAIP2 interact with U1 small nuclear ribonucleoprotein. This interaction enables U1 snRNPs to bind Pol II at parental gene promoters, regulating transcriptional activation of their respective parental genes (35). These nuclear circRNAs not only regulate transcription but also participate in chromatin looping and alternative splicing, further highlighting their multifaceted roles in gene expression dynamics (46).

Despite their evolutionary conservation and diverse biological roles, research into circRNAs is significantly hindered by several technical challenges. A primary obstacle is the accurate detection and identification of circRNAs, given their low abundance and the interference from highly similar linear RNA isoforms that share identical exonic sequences. This complexity extends to computational analysis, where large variations among sequencing algorithms complicate reliable back-splice junction identification and necessitate extensive molecular validation. Further compounding these issues are inconsistencies in circRNA nomenclature, with many circRNAs referred to by multiple names in the literature, often lacking standardized reporting of genomic positions or unique identifiers. This lack of a unified naming convention can hinder data comparison, reproducibility, and the efficient aggregation of research findings across studies. Furthermore, experimental validation methods like knockdown and overexpression are not easily scalable for large datasets generated. Encouragingly, recent advancements in long-read and single-cell RNA sequencing, coupled with deep learning algorithms, have significantly improved detection capabilities. Emerging DNA self-assembly technologies specifically designed for back-splice junction recognition and signal amplification also offer promising avenues for direct circRNA detection. Despite these innovations, the overarching difficulty in isolating high-purity circRNAs and precisely targeting their unique circular structure, without affecting linear counterparts, continues to limit the comprehensive exploration of their functions, particularly outside established research areas like cancer, cardiac, and neurodegenerative diseases. Nevertheless, fueled by recent insights into their biogenesis and biofunction, and recognizing their superior stability, tissue specificity, and distinct immunogenicity profiles, the pharmaceutical industry is actively exploring circRNAs not only as potential biomarkers but also as promising therapeutic tools for applications like vaccines, gene therapy, and protein replacement.

## 3 CircRNAs in skeletal muscle and immune regulation

### 3.1 Dynamic regulation of myogenesis, muscle stem cell function, and regeneration by circRNAs in muscle

In muscle tissue, circRNAs are dynamically regulated and play crucial roles during myogenesis, muscle stem cell (MuSC) function, and regeneration (14, 47, 48). Myogenesis, the formation of skeletal muscle, is a tightly orchestrated process involving myoblast proliferation, followed by their differentiation and fusion into multinucleated myotubes, the precursors of mature muscle fibers (49). CircRNAs exert complex control over these myogenic transitions, ensuring a sufficient pool of precursor cells and their timely commitment to forming functional muscle tissue (14).

Across diverse mammalian cell types, cell cycle progression and exit are controlled by shared molecular mechanisms. Central to this control is the cyclin-dependent kinase (Cdk) network, where orderly progression through the cell cycle is driven by five essential cyclin/Cdk complexes: cyclin D/Cdk4–6, cyclin E/Cdk2, cyclin A/Cdk2, cyclin A/Cdk1, and cyclin B/Cdk1. Multiple signals could influence the Cdk network to induce cell cycle arrest or proliferation (50). Several circRNAs have been identified that primarily influence the proliferation of myoblasts. To date, the most studied mechanism for these circRNAs is primarily through miRNA interactions that liberate proliferative signals, such as cyclins and B-cell lymphoma 2 (Bcl-2) from miRNA-mediated repression. circINSR regulates cell proliferation and apoptosis of bovine myocytes through removing the inhibition of miR-34a on Bcl-2 and CyclinE2 expression (51). circRBFOX2 was also confirmed to bind to miR-1a-3p and miR-206, both known to be involved in skeletal muscle development. Further validation suggested that circRBFOX2 antagonizes the functions of miR-206 in chicken myoblasts, leading to an upregulation of CyclinD2 mRNA and promotion of myoblast proliferation (52). Beyond typical miRNA regulatory roles, non-miRNA mechanisms for circRNAs functionally impact myogenesis. Notably, circ-ZNF609 impacts human and mouse myoblast proliferation via its translated protein. This circRNA contains an open reading frame, created upon circularization, and is translated through a splicing-dependent, cap-independent process, thereby demonstrating circRNA’s capacity as a protein-coding RNA with direct functional consequences in mammals (53).

Alongside the circRNA-mediated enhancement of proliferation, a critical aspect of maintaining the myoblast pool is the active suppression of myogenic differentiation factors like myoblast determination protein 1 (MyoD) and myocyte enhancer factor-2 (MEF2). The activity of MyoD and MEF2 is regulated by epigenetic modifications. MEF2 activity is repressed by its association with histone deacetylases HDAC4 and HDAC5, preventing MEF2 from initiating transcription, thereby inhibiting myogenic differentiation, and premature activation of muscle-specific gene programs (50). Experiments show that circLMO7 is able to bind and downregulate miR-378a-3p, thereby upregulating HDAC4, MyoD and MyoG to promote proliferation and inhibit differentiation in bovine myoblasts (54). circPAPD7 promotes proliferation and suppresses differentiation in goat MuSCs. Experimentally, it was shown that circPAD7 is able to counteract the inhibitory effect of miR-26a-5p by trapping it, thereby causing an increase in PAX7 and PCNA expression, a decrease in MyoD and MyoG, and subsequently relieving the suppression of Enhancer of zeste homolog 2 (EZH2). EZH2, a crucial epigenetic regulator and a component of the Polycomb Repressive Complex 2 (PRC2), is known for its role in maintaining stem cell self-renewal and proliferation, partly by repressing differentiation-specific genes in MuSCs (55). A similar pro-proliferative and anti-differentiative role is executed by a peptide circFAM188B-103aa, translated from circFAM188B (56). This protein interacts with Ribosomal Protein L4 (RPL4). This interaction ultimately modulates the cell cycle, leading to increased proliferation and inhibiting differentiation of chicken MuSC.

Several circRNAs modulate protein kinase B (AKT) signaling pathway, which controls essential cellular processes like survival, growth, and proliferation through the phosphorylation of its downstream targets, such as the Forkhead box O (FoxO) transcription factor, which controls myoblast proliferation, differentiation, and even muscle fiber type transformation (14, 20). Concurrently, the PI3K-AKT pathway is a central promoter of protein synthesis and a key activator of satellite cell proliferation, thereby critically supporting muscle repair and regeneration (49). circHUWE1 was found to promote cell proliferation and inhibit differentiation. Experimental work suggested that circHUWE1 sequesters miR-29, thereby reversing miR-29-mediated degradation or translational repression of AKT3 in bovine myoblasts. This mechanism contributes to the overall activation of the AKT signaling pathway (57).

Once a sufficient pool of cells has been generated, they eventually undergo a gradual process of specialization. Cell fate decisions, including proliferation, quiescence, differentiation, or apoptosis, are tightly controlled by both extracellular signals (e.g., mitogens, growth factors, Notch, Wnt/Wg, hedgehog, and TGF-β (Transforming growth factor-β), BMPs (bone morphogenetic proteins)) and cell-intrinsic transcription regulators that impact the cell cycle machinery. Developmental transitions involve coordinated events like activating cell cycle inhibitors and recruiting chromatin modifiers, ultimately leading to cell cycle arrest and the expression of genes specific to the differentiated cell type (50).

A distinct group of circRNAs primarily drives the transition towards myoblast differentiation, employing at least three recognized mechanisms: miRNA sponging, protein-mediated interactions, or direct protein production.

The first mechanism involves circRNAs acting as sequesters of specific miRNAs, which consequently de-repress the expression of pro-myogenic target genes or activate key signaling pathways. A notable example is CDR1as (also known as ciRS-7), which enhances goat MuSC differentiation by interacting with miR-7. This sequestration of miR-7 leads to increased levels of its target, Insulin-like Growth Factor-1 Receptor (IGF1R), a key promoter of differentiation. Illustrating a sophisticated regulatory network, the transcription of CDR1as itself is activated by MyoD, which binds to a canonical E-box in the CDR1 promoter, forming a positive feedback loop that reinforces the myogenic program (58). Other validated findings include bovine circFGFR4 (59) and circSNX29 (60) that promote differentiation by modulating Wnt signaling. Specifically, circFGFR4 promotes myoblast differentiation by binding miR-107 to relieve its inhibition of Wnt3a/β-catenin, inducing expression of Myosin heavy chain (MyHC), MyoD, and Myogenin (MyoG), while circSNX29 directly interacts with and downregulates miR-744, thereby activating the Wnt5a/Ca^2+^ signaling pathway via CaMKIIδ (Calmodulin kinase)/NFATC1 (nuclear factor of activated T-cells, cytoplasmic 1) activation of myogenic genes. Chicken and mouse circMEF2A1 also promote differentiation by regulating the miR-30a-3p/PPP3CA (Protein Phosphatase 3 Catalytic Subunit Alpha)/NFATC1 axis, and circMEF2A2, which targets the miR-148a-5p/SLIT3 (Slit Guidance Ligand 3)/ROBO2 (Roundabout Guidance Receptor 2)/β-catenin signaling pathway (61). Similarly, in goats, circTGFβ2 was experimentally shown to promote myoblast differentiation by interacting with miR-206 and miR-211, which alleviate their suppression of crucial myogenic markers like MyoD, MyoG, and MyHC (62).

Beyond miRNA binding, some circRNAs facilitate differentiation through more direct protein-level interactions or even by being translated into functional proteins themselves. For instance, circMYBPC1 (63) provides an example of direct protein interaction by binding to the Myosin Heavy Chain (MyHC) protein. This interaction increases MyHC expression at both mRNA and protein levels, thereby promoting cell differentiation in cattle (this finding was subsequently validated *in vivo* using a mouse cardiotoxin injury model, which is discussed further below). In another example, circSmad4, which is overexpressed during mouse skeletal muscle differentiation, enhances MyHC production by reducing the binding of Purine Rich Element Binding Protein A (PURA) and B (PURB) (which normally repress MyHC) at the MHC promoter (43).

Further, porcine circKANSL1L (64) is translated into a functional protein able to directly interact with AKT, promoting the phosphorylation of FoxO3 and ultimately activating the AKT-FoxO signaling pathway to drive differentiation.

A subset of circRNAs demonstrates a sophisticated capacity to promote both the proliferation and subsequent differentiation of myoblasts, highlighting their role in orchestrating these distinct cellular states. The transition from proliferation to differentiation is a highly coordinated process, requiring extensive modifications to the cell’s transcriptome, epigenetic landscape, and chromosome architecture. Central to this cell fate decision-making are transcriptional master regulators, including the basic helix-loop-helix (bHLH), and myogenic regulatory factors (MRFs) and the MEF2 family. Several circRNAs exert their dual influence by modulating these key regulators or associated pathways, often shown in studies through miRNA sponging. The subsequent examples detail specific mechanisms where circRNAs target miRNAs, with these interactions experimentally validated. Studies, particularly in chicken models, have identified circRNAs’ modulation of MEF2 and bHLH factors. For example, circSVIL promotes both proliferation and differentiation by targeting miR-203, thereby regulating MEF2C enhancer factor (65). Similarly, circHIPK3 exhibits dual functionality. In one context, it sponges miR-30a-3p to upregulate MEF2C (66). In mouse C2C12 cells, circHIPK3 overexpression also reversed the inhibitory effect of miR-7 on both proliferation and differentiation by increasing the expression of Transcription Factor 12 (TCF12), a bHLH E-protein family member (67). In chicken MuSCs, circFNDC3AL enhances both processes by binding miR-204. This action upregulates BCL9, a critical co-activator for Wnt signaling and a regulator of differentiation-related genes (68).

CircRNAs also influence proliferation and differentiation by modulating growth factor-mediated signaling, which is crucial for both processes. For instance, bovine circTTN was shown to enhance both processes. Mechanistically, circTTN showed interaction with miR-432. This action increases the protein expression of Insulin-like Growth Factor-2 (IGF2) and key components of the PI3K/AKT signaling pathway, such as IRS1 (Insulin Receptor Substrate 1), PI3K (Phosphoinositide 3-kinases), PDK1 (Pyruvate Dehydrogenase Kinase 1), and AKT (69). Similarly, it was demonstrated that circUSP13 binds miR-29c, promoting Insulin-like Growth Factor 1 (IGF1) expression and PI3K/AKT activation, leading to MyoG and MyHC upregulation (70). CircPPP1R13B facilitates chicken MuSC proliferation and differentiation via targeting miR-9-5p. This de-represses IGF2BP3 (Insulin Like Growth Factor 2 MRNA Binding Protein 3) expression, consequently triggering the IGF/PI3K/AKT signaling pathway, which is vital for both cell growth and myogenic progression (71).

**Figure 1:**
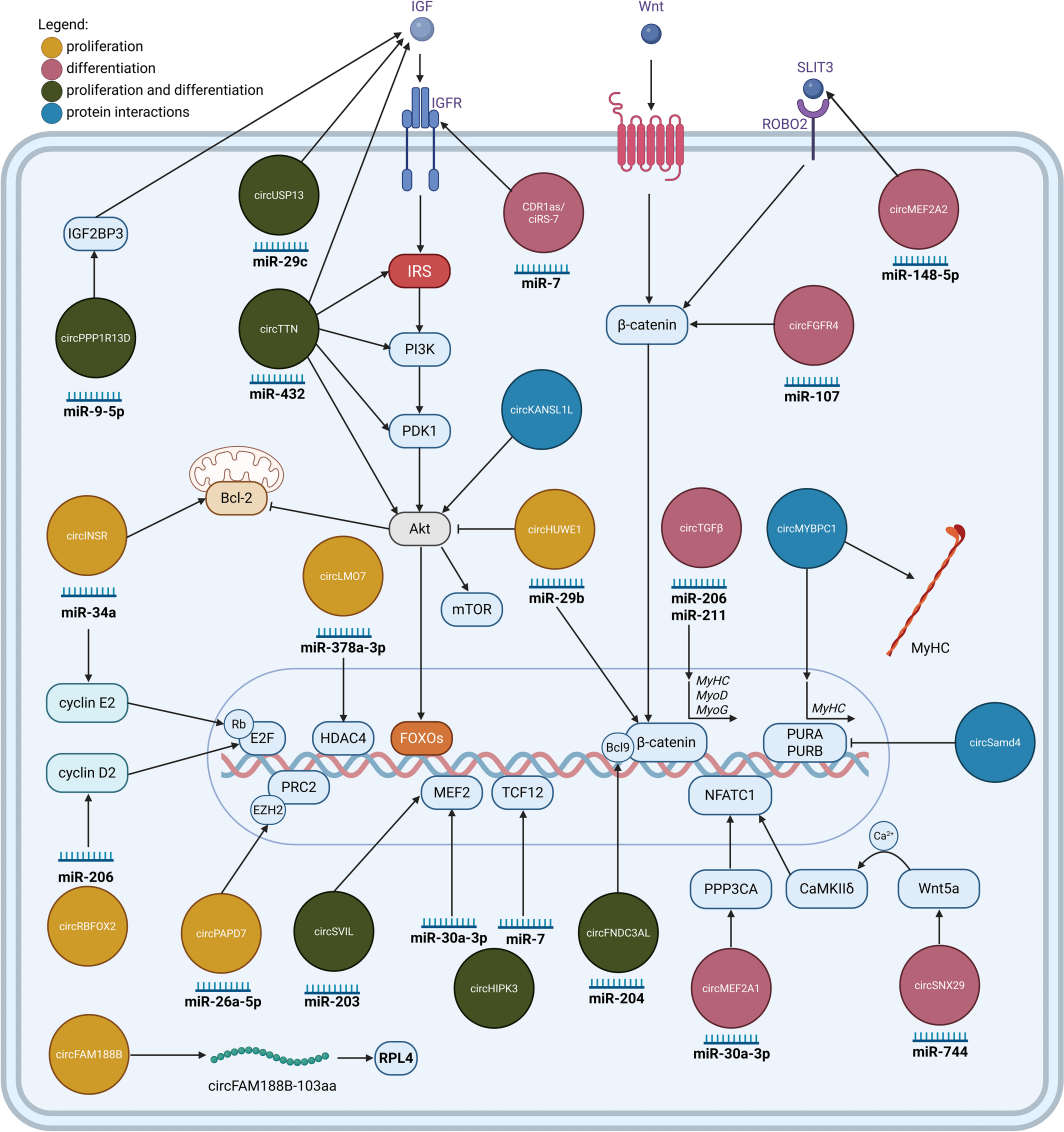
CircRNAs involved in the regulation of myogenesis. CircRNAs regulating myoblast proliferation (yellow), and differentiation (pink), or both processes (green). Most circRNAs function as molecular sponges for specific miRNAs, inhibiting their regulatory effect. Exceptions include circFAM188B, which encodes a protein, and the circRNAs which exert their function by binding to proteins and modulating their activity (blue). Illustration was created with BioRender.com.

Summarized findings of circRNAs regulation of myogenesis and regeneration in muscle are presented in **Figure 1**.

(For a comprehensive overview of functional circRNAs in myogenesis, readers are encouraged to refer to the review by Sun et al., 2022 (14) and Huang et al., 2025 (20)).

#### 3.1.1 Skeletal muscle regeneration after muscle injury

**Figure 2:**
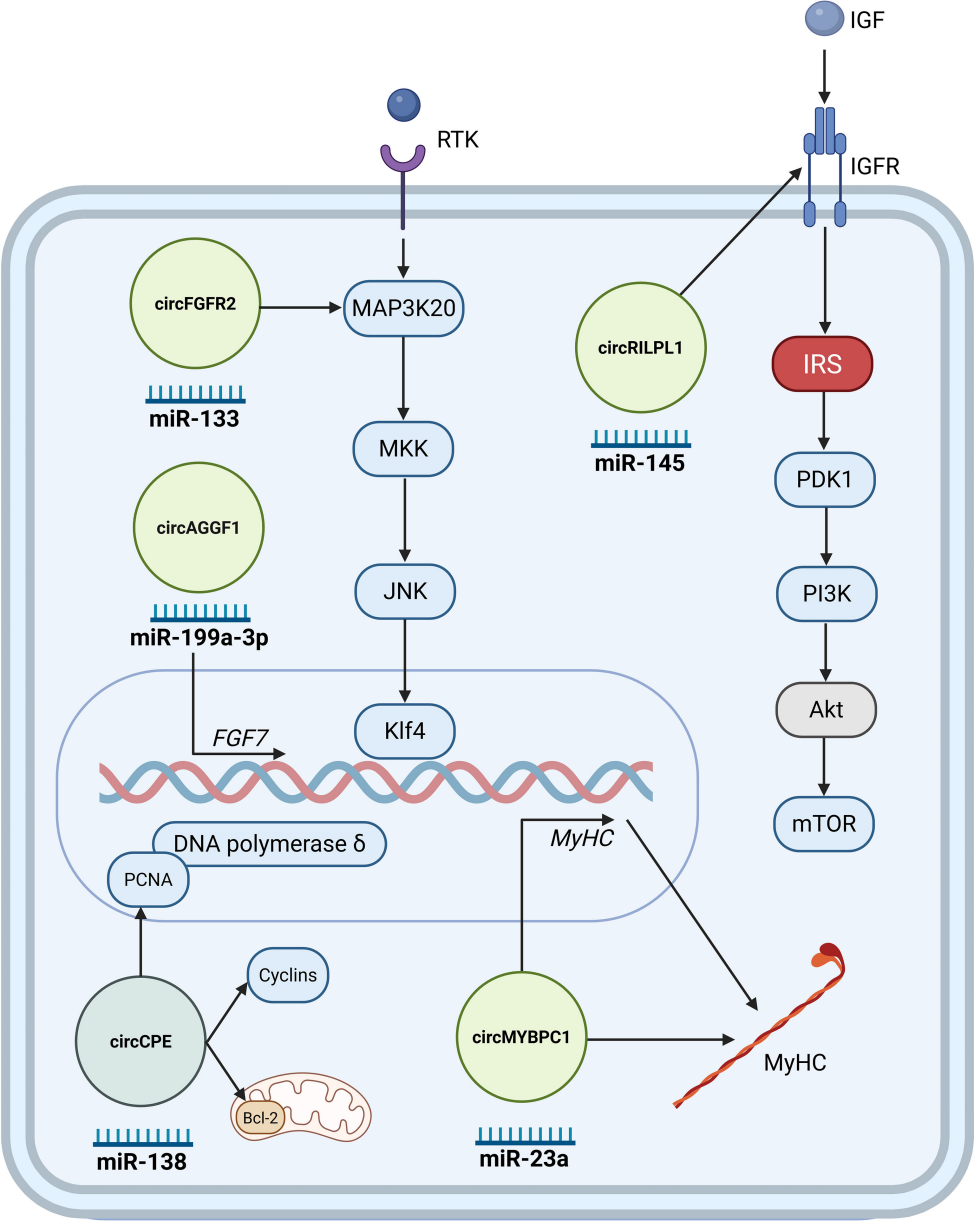
Key circRNAs regulating injury-induced regeneration. CircRNAs promoting regeneration are shown in light green, whereas the circRNAs impairing regeneration in a dark green circle. These circRNAs typically exert their effects through miRNA sponging, influencing the fate and activity of satellite cells during muscle regeneration. Illustration was created with BioRender.com.

Studies have demonstrated that various circRNAs play significant roles in muscle regeneration (**Figure 2**). Utilizing cardiotoxin skeletal muscle injury mouse models, studies validate the circRNA involvement in muscle regeneration. circFgfr2 promotes muscle regeneration primarily by acting as a decoy for miR-133. This sequestration alleviates the suppression of miR-133’s target, Map3k20, leading to the activation of the JNK/MAPK signaling pathway that activates transcription factor Klf4, ultimately contributing to cell differentiation and regeneration (21). Several other circRNAs such as circMYBPC1 (63), circRILPL1 (72), and circAGGF1 (73) may stimulate skeletal muscle regeneration in injured mouse muscle. *In vitro*, circMYBPC1 promotes myoblast differentiation by sequestering miR-23a, thereby relieving the miRNA’s inhibitory effect on the expression of MyHC. Mechanistically, circMYBPC1 positively upregulated the expression of myosin heavy chain (MyHC) by directly interacting with miR-23a and binding MyHC protein (63). circRILPL1 promotes muscle regeneration by targeting miR-145, which influences IGF1R levels and subsequently activates the PI3K/AKT pathway which in turn induces myoblast proliferation and differentiation (72). circAGGF1 regulates myogenic processes via sequestration of miR-199a-3p, which prevents it from suppressing Fibroblast Growth Factor 7 (Fgf7), leading to increased Fgf7 levels, subsequently promoting upregulation of canonical myogenic markers (73). Conversely, some circRNAs impair muscle regeneration. In a cardiotoxin induced injury mouse model, circCPE overexpression was shown to attenuate muscle repair. Mechanistic studies in bovine myoblasts revealed that overexpressing circCPE promotes cell proliferation (by increasing markers like Proliferating Cell Nuclear Antigen and cyclins) and inhibits apoptosis (by increasing Bcl-2) by diminishing the effect of miR-138 on both processes. This combined effect, forcing proliferation while blocking differentiation, ultimately leads to defective muscle regeneration (74).

The accumulating evidence demonstrates the multifaceted involvement of circRNAs in muscle development, stem cell biology, and tissue repair, showcasing their diverse regulatory mechanisms including miRNA sequestration, protein interaction, and translation. Despite the robust insights gained from numerous animal model studies, a critical gap remains in extrapolating these findings directly to humans. Although many circRNAs exhibit high sequence conservation across species, rigorous validation studies in human muscle tissues and stem cells are urgently needed to confirm the precise functional implications and mechanistic details in our species.

### 3.2 Muscle wasting

#### 3.2.1 Circular RNAs modulating innate immunity induced muscle wasting

In muscle immune-related diseases, innate immunity is activated when damage-associated molecular patterns released from injured cells activate Toll-like receptors (TLRs), triggering the production of pro-inflammatory cytokines like Tumor Necrosis Factor (TNF-α) and Type I interferons. These cytokines directly induce muscle cell death and activate the NF-κB pathway, which exacerbates damage by blocking new muscle formation via MyoD inhibition (75). Concurrently, non-immune mechanisms, such as MHC class I overexpression, induce endoplasmic reticulum stress and an overload response, which also activates NF-κB and downstream caspases, leading to apoptosis (75).

Several circRNAs have been identified that modulate this pathway. CircAGO3, derived from the AGO3 gene, is highly expressed in atrophying chicken muscle and interacts with miR-34b-5p, relieving inhibition on TNF Receptor Associated Factor 3 (TRAF3), an upstream activator of NF-κB signaling (15). Enhanced NF-κB activity increases expression of atrophy markers, linking circAGO3 to inflammation-driven muscle atrophy (15). Another recently discovered circRNA, circTmeff1 (76), found in a mouse atrophy model, promotes muscle loss by binding to the RNA-binding protein TDP-43 and sequestering it in mitochondria, which triggers release of mitochondrial DNA and activation of the cGAS-STING innate immune pathway, which again leads to NF-κB and interferon signaling that exacerbates muscle protein breakdown (77). Knockdown of circTmeff1 blunts NF-κB–associated gene induction and partially rescues muscle mass in diverse atrophy models. These examples underscore that circRNAs can amplify NF-κB catabolic signaling by miRNA sponging (in the case of circAGO3) or by aberrant protein/RNA interactions (in the case of circTmeff1), thereby driving inflammatory muscle atrophy.

#### 3.2.2 Circular RNAs modulating adaptive immunity induced muscle wasting

Moreover, adaptive immune mechanisms contribute to muscle pathology by modulating key pathways involved in fibrosis and wasting (78). Chronic production of TGF-β by immune cells drives fibrosis and preclinical models have shown that inhibiting the signaling of TGF-β and the related protein myostatin is a potent strategy for mitigating muscle fibrosis and improving muscle function (79).

Myostatin, a TGF-β family cytokine, is a master negative regulator of muscle mass, and it signals through activin receptor type IIB (ActRIIB) to phosphorylate Smad2/3 transcription factors, which induce atrophy genes (such as Atrogin-1) and inhibit muscle protein synthesis (80). In atrophic chicken muscle circTMTC1 is highly expressed (18). This circRNA contributes to muscle loss by inhibiting myoblast differentiation through circTMTC1/miR-128-3p/myostatin axis, which effectively increases myostatin levels and induces muscle mass loss (18). Conversely, circANAPC7 ameliorates muscle wasting in a human pancreatic cancer cachexia model. The anti-atrophic effect of circANAPC7, further validated in a mouse model, showed it functions by trapping miR-373 (which is induced by cachectic factors). This trapping prevents miR-373 from downregulating PHLPP2, a phosphatase that activates AKT (81). The restoration of AKT activity by circANAPC7 leads to a reduction in muscle proteolysis. In addition, circANAPC7’s action was found to dephosphorylate and inactivate STAT5 (Signal Transducer and Activator of Transcription 5), which in turn reduced the secretion of TGF-β from muscle (81). In summary, the myostatin pathway has complex circRNAs regulation, but manipulating them may offer new strategies to blunt myostatin’s involvement in various muscle-related disorders (80).

#### 3.2.3 Circular RNAs modulating glucocorticoid-induced muscle atrophy (dexamethasone treatment)

Glucocorticoids are commonly used in immune-directed therapy in idiopathic inflammatory myopathies (82), Duchenne muscular dystrophy (83), and myasthenia gravis (84) due to their ability to suppress detrimental inflammatory and immune responses, and to inhibit the NF-κB pathway, which is upregulated in patients. This inhibition of NF-κB presumably counteracts the catabolic effects of this pathway on muscle (85). However, a primary adverse effect of glucocorticoids is the induction of muscle atrophy. Furthermore, they can hinder muscle regeneration by inhibiting myogenic cell proliferation and differentiation (85).

Glucocorticoid-induced muscle catabolism is mediated by several interconnected mechanisms. Firstly, it involves the activation of the ubiquitin-proteasome pathway through the inhibition of PI3K-AKT signaling (86). This inhibition leads to increased FoxO transcription factor activity and the subsequent transactivation of E3 ubiquitin ligases MAFbx and MuRF1, which target sarcomeric proteins like MyHC for degradation (87). Secondly, glucocorticoids induce anabolic resistance to regulators like IGF-1 and insulin, inhibit amino acid transport into muscle (notably glutamine), and repress AKT–GSK3β–β-catenin activity. Finally, glucocorticoids can upregulate myostatin, which inhibits the AKT-mTORC1 protein synthesis pathway via Smad2/3 activation (88). In models of glucocorticoid-induced atrophy, such as C2C12 myotubes treated with dexamethasone, circSmox is notably upregulated (89). While its downstream targets are still being fully elucidated in this specific context, this upregulation of circSmox is accompanied by co-elevated levels of p21 mRNA (89). Given that p21 is a well-known cell cycle inhibitor, its increased expression is consistent with a decrease in cell proliferation, a characteristic feature observed in glucocorticoid-induced muscle atrophy.

circCCDC91 was identified in chicken muscle as downregulated under dexamethasone treatment. The overexpression of circCCDC91 significantly alleviated the atrophic effects induced by dexamethasone treatment in this model. Mechanistically, circCCDC91 could act as a molecular sponge for the miR-15 family, leading to the upregulation of IRS1. This in turn reactivated IGF-1–PI3K–AKT signaling, thereby attenuating muscle atrophy (90).

#### 3.2.4 Multiple atrophy models

A striking example of a circRNA driving multiple mediated atrophy is circDdb1, which is upregulated across multiple atrophy models in mice. Functionally, overexpression of circDdb1 is sufficient to cause muscle fiber atrophy, whereas silencing circDdb1 mitigates muscle wasting induced by Ang II, TNF-α, or dexamethasone treatment (91). Experimentally, circDdb1 could undergo “rolling translation” to produce an 867-amino acid protein (circDdb1-867aa) (91). This protein was found to bind to eukaryotic elongation factor 2 (eEF2) and enhance its phosphorylation at Thr56, an inhibitory modification that decreases cellular protein translation, tipping the balance toward loss of muscle mass. Similarly, the involvement of the aforementioned circTmeff1 was also confirmed in several *in vitro* models of muscle atrophy, including those induced by dexamethasone treatment, TNF-α, or Ang II treatment (76).

**Figure 3:**
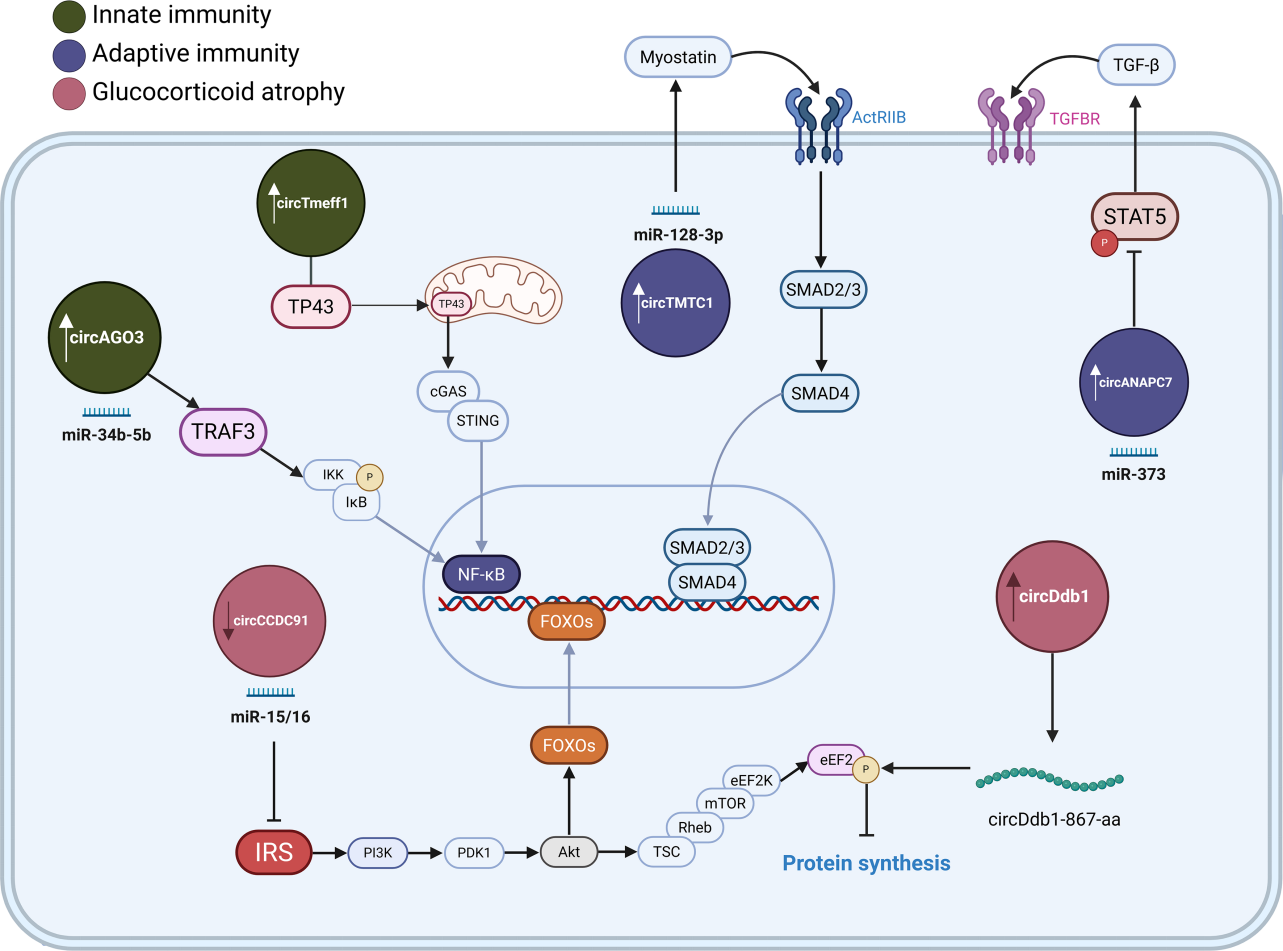
CircRNAs associated with muscle atrophy. CircRNAs involved in immune-mediated muscle atrophy trough innate (green) and adaptive immunity (purple circles). circRNAs are involved in glucocorticoid-induced muscle atrophy (pink). Arrows indicate their upregulation or downregulation during muscle-wasting. These circRNAs affect protein metabolism by sponging miRNAs, binding signalling proteins or by being translated into proteins, contributing to the dysregulation of NF-κB, TGF-β and PI3K/AKT signalling pathways. Illustration was created with BioRender.com.

Summarized findings of circRNAs’ involvement in muscle wasting are presented in **Figure 3**.

In conclusion, the current body of research unequivocally positions circRNAs as potent regulators across the spectrum of muscle wasting animal models, spanning innate and adaptive immunity-induced atrophy, as well as glucocorticoid exposure. While compelling evidence suggests that certain circRNAs, exemplified by circDdb1 and circTmeff1, may exert influence across multiple catabolic conditions, a comprehensive understanding necessitates discerning whether these involve distinct, model-specific circRNA-mediated interactions and their precise underlying mechanisms, or if these circRNAs predominantly converge on shared catabolic signaling nodes. Therefore, validating whether these specific circRNA-mediated catabolic pathways, identified here in animal models, are conserved and functionally active in human myopathies is the critical next step toward clinical translation.

### 3.3 CircRNAs in immune cells driving inflammation

The nature and location of inflammatory infiltrates are key distinguishing features among various immune-related muscular diseases. In idiopathic inflammatory myopathy, the patterns are subtype-specific: dermatomyositis shows perivascular/perimysial infiltrates of mixed immune cells; polymyositis has endomysial T-cells; immune-mediated necrotizing myopathy features macrophage-predominant infiltrates; antisynthetase syndrome has scattered perimysial infiltrates; and inclusion body myositis is defined by endomysial CD8^+^ T-cells invading non-necrotic fibers (2). In Duchenne muscular dystrophy, the inflammation is a secondary response to muscle damage, dominated by macrophages and, later, CD4^+^ and CD8^+^ T-cells, which drive fibrosis (92). In contrast, myasthenia gravis lacks a primary muscle infiltrate; its pathology is driven by circulating autoantibodies and activated T/B cells from the blood, which cause damage at the neuromuscular junction, attracting a localized secondary infiltrate of macrophages (93).

#### 3.3.1 CircRNA expression in lymphocytes

T-cells and B-cells, as central players in adaptive immunity, are key drivers of autoimmune diseases. Emerging evidence reveals that these lymphocytes are also subject to intricate regulation by circRNAs, which can significantly influence their development, activation, differentiation, and effector functions, thereby impacting autoimmune pathology. A comprehensive study by Gaffo et al., 2019 (24) provided a foundational catalogue of circRNAs in B-cells, T-cells, and monocytes isolated from peripheral blood of healthy adult donors, revealing distinct cell-type specific expression patterns and alternative circularization events. In T-cells circIKZF1, circTNIK, circTXK, and circFBXW7 were found to be overexpressed, while in B-cells circPAX5, circAFF3, circIL4R, and circSETBP1 display distinct expression patterns, assisting in the regulation of differentiation and activity. For example, circPAX5, originating from a key B-lineage transcription factor, is essential for controlling B-lymphocyte differentiation and preserving B-cell identity, potentially by influencing gene expression patterns unique to B-cell maturation. Likewise, circIL4R may contribute to B-cell activation and enhance the immune response in conjunction with interleukin receptor signaling (24).

Pro-inflammatory T helper cell subsets, such as Th1 and Th17 cells, contribute to autoimmunity by producing potent cytokines like Interferon gamma (IFN-γ) and IL-17, respectively, which amplify local inflammation and tissue damage. Recent studies indicate that specific circRNAs can promote these pathogenic T-cell responses. For instance, circNUP214 is upregulated in the CD4^+^ T-cells of rheumatoid arthritis patients and has been shown to promote Th17 differentiation (94). Similarly, circINPP4B enhances Th17 cell differentiation and progression of autoimmune encephalomyelitis by activating the circINPP4B/miR-30a/IL-21R axis (95).

#### 3.3.2 CircRNA expression in macrophages

Experimentally validated in mice circRNA Cdr1as regulates macrophage plasticity. It was found to be downregulated in pro-inflammatory M1 macrophages while overexpression of circRNA Cdr1as acts as an anti-inflammatory brake in macrophages and skews macrophages toward the M2 phenotype, fosters an anti-inflammatory, pro-regenerative microenvironment (96). In contrast, circHIPK3 overexpression in macrophages exemplifies a pro-inflammatory role, by interacting with miR-192 and miR-561. Downregulation of miR-561 significantly upregulated NLR Family Pyrin Domain Containing 3 (NLRP3) expression, thereby enhancing the assembly and activation of the NLRP3 inflammasome, resulting in heightened release of inflammatory mediators (97). Similarly, circPPM1F has been identified as a positive regulator of classically activated (M1) macrophages via stabilization of NF-κB signaling components (98). A recent study investigating global circRNA expression patterns during M1 (IFN-γ/LPS stimulated) versus M2 (IL-4 stimulated) polarization of the human THP1 cell lines identified circRNF19B (hsa_circ_0000048) as significantly upregulated in the M1 phenotype (23). Potential mechanism for circRNF19B’s activation of macrophage polarization towards the pro-inflammatory M1 phenotype could be trough miRNA-mediated interaction with macrophage plasticity (23).

Beyond polarization, circRNAs regulate other key immune functions like cell trafficking. A key example is the LPS-inducible mouse circRNA mcircRasGEF1B, which is co-transcriptionally induced with its linear mRNA by TRL agonists. Mechanistically, mcircRasGEF1B exerts its function through maintaining the stability of mature ICAM-1 mRNA, a crucial adhesion molecule. Given ICAM-1’s importance in leukocyte recruitment and antigen presentation, this circRNA-mediated stabilization of its mRNA highlights a novel mechanism by which circRNAs can influence innate immune responses and inflammatory conditions (99).

Although not yet specifically studied in the context of idiopathic inflammatory myopathies and Duchenne muscular dystrophy, it is plausible that analogous circRNAs could fuel the accumulation and activity of Th1 and/or Th17 cells within affected muscle tissue, thereby elevating local IL-17 and IFN-γ levels and contributing to myofiber damage. Furthermore, broader transcriptomic analyses in systemic autoimmune conditions have revealed that dozens of circRNAs in PBMCs are dysregulated during active inflammation (8), highlighting a complex layer of circRNA-mediated immune modulation that warrants further investigation to fill existing gaps in our understanding.

## 4 Potential implications of circRNAs in specific muscular immune-related diseases

### 4.1 Idiopathic inflammatory myopathies

Idiopathic inflammatory myopathies represent a heterogeneous group of autoimmune disorders characterized by chronic muscle inflammation, progressive muscle weakness, and often extra-muscular manifestations such as interstitial lung disease and arthritis (2). The underlying pathology involves complex interactions between immune cells, muscle cells, and the local microenvironment, leading to muscle damage and dysfunction. Idiopathic inflammatory myopathies can be classified into several subgroups, including dermatomyositis, polymyositis, anti-synthetase syndrome, immune-mediated necrotizing myopathy, inclusion body myositis, and overlap myositis (2). Apart from muscle involvement, which is particularly pronounced in immune−mediated necrotizing myopathy and inclusion−body myositis, the clinical features of dermatomyositis, polymyositis, and anti−synthetase syndrome commonly include systemic manifestations that affect the skin, lungs, joints, cardiovascular system and/or gastrointestinal system. Research indicates that distinct gene expression profiles, reflecting specific underlying cellular and tissue regulation, can differentiate between disease subtypes even when histological patterns within muscle tissue appear similar, highlighting the molecular heterogeneity of idiopathic inflammatory myopathiess. For example, the expression of type I interferon-inducible genes is markedly higher in dermatomyositis compared to anti-synthetase syndrome (100, 101). Indeed, molecular signatures are so pronounced that transcriptomic data from muscle biopsies can, on its own, accurately classify samples from patients with different types of idiopathic inflammatory myopathies with greater than 90% accuracy (102).

#### 4.1.1 Dysregulated circRNA expression profiles in idiopathic inflammatory myopathies

Although specific, large-scale studies directly addressing the comprehensive circRNA landscape across all idiopathic inflammatory myopathy subtypes are still lacking, initial insights can be drawn from broader myopathy research. A study by Tsitsipatis et al., 2022 (103), while focused on amyotrophic lateral sclerosis, included muscle biopsy samples from healthy controls and myopathy patients (Inflammatory myopathies, n = 5; Mitochondrial, n = 2; Inclusion Body myositis, n = 1) as disease controls. The authors sequenced ALS samples and identified 50 candidate circRNAs and confirmed 8 were upregulated and 10 downregulated via PCR. Myopathy and neuropathy samples were then used to identify common mechanisms in neuromuscular diseases. Testing these 18 circRNAs found to be deregulated in ALS, in myopathy samples compared to healthy controls 6 were downregulated (circITGB6 (hsa_circ_0056856), circZCCHC2 (hsa_circ_0047886), circALPK2 (hsa_circ_0141401), circNPHP1 (hsa_circ_0117010), circCCDC9 (hsa_circ_0000944), and circZNF362 (hsa_circ_0009027)) and 2 upregulated (circAMY2B (hsa_circ_0000099) and circARHGAP12 (hsa_circ_0000231)). Thus, while the study finds some common dysregulated circRNAs in neuromuscular diseases, due to its study design, it fails to identify most changed circRNAs in myopathies. These observations suggest that distinct circRNA expression profiles may contribute to the molecular landscape in myopathy; however, this finding needs to be validated in further larger patient cohorts.

#### 4.1.2 Potential functional roles and mechanistic insights of specific circRNAs (hypotheses derived)

While the observed dysregulation of circRNAs in myopathy samples does not inherently confirm their functional importance in idiopathic inflammatory myopathies pathogenesis, the potential relevance of these expression changes to idiopathic inflammatory myopathies pathology remains entirely speculative and requires further validation. The following hypotheses regarding potential circRNA mechanisms in muscle are currently largely theoretical, drawing upon their reported roles in other biological systems (**Supplementary List L1**). For instance, circRNAs have been shown to affect critical muscle repair molecules like TGF-β signaling; inhibit developmental signaling pathways such as Wnt and NOTCH1; control the expression of surface differentiation markers like Caveolin 1 (CAV1) and Podoplanin (PDPN); and influence macrophage and T-cell polarization and angiogenesis in various cell types, including cancer, endothelial, and epithelial cells. These extrapolations require rigorous experimental validation directly within idiopathic inflammatory myopathies-specific models and patient cohorts. The current understanding of circRNAs in idiopathic inflammatory myopathies is nascent. Future research is needed, encompassing not only muscle biopsies but also sorted immune cell populations and serum/plasma.

### 4.2 Duchenne muscular dystrophy

Duchenne muscular dystrophy is a severe X-linked recessive genetic disorder caused by mutations in the dystrophin gene, leading to the absence or dysfunction of the dystrophin protein (3). This fundamental defect results in progressive muscle degeneration, chronic inflammation, fibrosis, and impaired muscle regeneration, ultimately causing loss of ambulation and premature death. While the primary genetic cause is well-established, the complex molecular sequelae contributing to disease progression are still being actively investigated (3). Emerging research highlights significant alterations in the expression and function of circRNAs in Duchenne muscular dystrophy, suggesting their involvement in the disease’s multifaceted pathology.

#### 4.2.1 Dysregulated circRNA expression profiles in Duchenne muscular dystrophy – human and animal models

Legnini et al., 2017 (53) investigated circRNA expression during the *in vitro* differentiation of human myoblasts from Duchenne muscular dystrophy patients versus healthy controls. They report unique circRNA expression profiles in the disease, suggesting potential disruption in the competitive biogenesis and balance between circular and linear RNA isoforms. Complementing these human studies, comprehensive analysis of circRNA expression in the muscle of mdx mice, a common animal model for Duchenne muscular dystrophy, compared to C57 healthy controls, has identified 197 differentially expressed circRNAs (94 upregulated and 103 downregulated). Gene Ontology (GO) and KEGG pathway analyses of the host genes for this entire set implicated them in relevant processes like muscle structure development, and in cAMP and calcium signaling pathways, which are known to be dysregulated in Duchenne muscular dystrophy (104).

#### 4.2.2 Potential functional roles and mechanistic insights of specific circRNAs in Duchenne muscular dystrophy

Among specific circRNAs, circ-ZNF609 has emerged as particularly interesting, as Legnini et al., 2017 (53) found it to be aberrantly elevated and failed to be downregulated during differentiation in Duchenne muscular dystrophy myoblasts. This persistent high expression correlates with impaired myogenic differentiation and sustained proliferation of Duchenne muscular dystrophy myoblasts. The pro-proliferative effect of elevated circ-ZNF609 in Duchenne muscular dystrophy may be mediated by its influence on cell cycle regulators (e.g., CDK1, Cyclin A2). This study provided evidence that circ-ZNF609 can be translated into a protein; however, while the precise function of the circ-ZNF609-encoded protein in Duchenne muscular dystrophy pathology requires further elucidation, its nuclear localization suggests it could directly influence gene expression or other nuclear processes related to myoblast fate. The sustained expression of this normally pro-proliferative factor could contribute to the defective differentiation and potentially the exhaustion of the satellite cell pool observed in Duchenne muscular dystrophy. The same study (53) also reported that circ-QKI and circ-BNC2 are significantly downregulated in primary myoblasts from Duchenne muscular dystrophy patients. In normal myogenesis, both of these circRNAs are known to be crucial promoters of myoblast differentiation. In line with this pro-differentiation role, their downregulation in Duchenne muscular dystrophy myoblasts correlates strongly with the observed delay in myogenic differentiation that is characteristic of the disease (**Table 1**, **Supplementary L1**).

**Table 1:** Summary of key dysregulated circRNAs in Duchenne muscular dystrophy (DMD).

circRNA (alias)	Participants /cell culture	Observed dysregulation	Primary proposed mechanism / role in disease context	Ref
circ-ZNF609	Human myoblasts (WT and DMD)	Persistently up-regulated in DMD myoblasts; fails to fall during differentiation	Encodes a protein that localises to the nucleus and promotes cell-cycle gene expression (e.g., CDK1, CCNA2). Sustained abundance keeps myoblasts proliferative and impairs their differentiation, potentially depleting the satellite-cell pool.	(53)
circ-QKI	Human myoblasts (WT and DMD)	Down-regulated in DMD myoblasts	Down-regulation correlates with delayed myogenic differentiation in DMD.	(53)
circ-BNC2	Human myoblasts (WT and DMD)	Down-regulated in DMD myoblasts	Similar to circ-QKI; reduced levels are linked to impaired differentiation of dystrophic myoblasts.	(53)
circHIPK3 (mmu_ circRNA_19008)	n = 3 mdx mice n = 3 C57 mice	Up-regulated in muscle from mdx mice	Over-expression may disturb miRNA networks that support muscle regeneration.	(104)
circRNA_36990 (Mpdz)	n = 3 mdx mice n = 3 C57 mice	Down-regulated in muscle from mdx mice	Bioinformatically predicted to encode a novel peptide; functional consequences still unknown but candidate contributor to dystrophic pathology.	(104)
circRNA_32522 (Ide)	n = 3 mdx mice n = 3 C57 mice	Down-regulated in muscle from mdx mice	Predicted protein-coding circRNA; putative roles in muscle degeneration/inflammation require validation.	(104)
circRNA_40856 (Erc1)	n = 3 mdx mice n = 3 C57 mice	Down-regulated in muscle from mdx mice	Predicted to translate; post-transcriptional regulation in the dystrophic muscle; functional studies pending.	(104)
circRNA_43272 (Zfp423)	n = 3 mdx mice n = 3 C57 mice	Up-regulated in muscle from mdx mice	Predicted protein-coding potential; post-transcriptional regulation in the dystrophic muscle; functional studies pending.	(104)
circRNA_19191 (Plcl2)	n = 3 mdx mice n = 3 C57 mice	Up-regulated in muscle from mdx mice	Predicted protein-coding potential; may participate in calcium/phospholipid signalling relevant to muscle fibre integrity; functional studies pending.	(104)

The Legnini et al. study (53) analysed primary human DMD myoblasts during proliferation and differentiation, whereas Song et al. (104) profiled diaphragm muscle from mdx mice—the standard DMD model—and applied in-silico open-reading-frame prediction. Reference (61, 62) provided mechanistic insight for circHIPK3; the other predicted coding circRNAs have not yet been functionally tested.

The finding that circ-ZNF609 can be translated into a functional protein has broadened our understanding of circRNA roles in Duchenne muscular dystrophy. Building on this concept, Song et al., 2019 (104) utilized bioinformatics in an mdx mice model of Duchenne muscular dystrophy to predict the protein-coding potential of other circRNAs dysregulated in dystrophic muscle. They analyzed, based on m^6^A motif and an open reading frame (ORF), a subset of circRNAs that could encode novel proteins. Among the top five predicted protein-coding candidates, three were downregulated, circMpdz (mmu_circRNA_36990), circIde (mmu_circRNA_32522), and circErc1 (mmu_circRNA_40856), and two were upregulated, circZfp423 (mmu_circRNA_43272) and circPlcl2 (mmu_circRNA_19191). A separate functional analysis of this potentially translatable subset suggested their involvement in different pathways, including metal ion binding, covalent chromatin modification, and cGMP-dependent protein kinase signaling. Notably, the study also suggested decreased overall m^6^A modification and the translation of circRNAs in the muscle of mdx mice compared to controls, indicating a broader disruption of post-transcriptional regulation in the dystrophic muscle. This is emerging evidence that these circRNA-derived proteins could be previously overlooked contributors to Duchenne muscular dystrophy pathogenesis, with potential roles in muscle degeneration, inflammation, or regeneration (**Table 1**). In this mdx mouse model study, circHIPK3 (circRNA_19008) was identified as the top upregulated (104), and while its direct mechanism in Duchenne muscular dystrophy from this specific study is less detailed, others (105) provided mechanistic insight, suggesting circRNA_19008 can potentially affect myogenesis by sponging miR-186-5p and promoting myoblast differentiation via targeting MyoG and MEF2A.

While the observed alterations in circRNA expression, and the predicted functional implications of their host genes and translated products, align well with known Duchenne muscular dystrophy pathologies (e.g., muscle structure, calcium/cAMP signaling, m^6^A modification), the field is now tasked with systematically validating the precise functional roles and downstream molecular consequences of these circRNAs and their protein products within the complex, evolving microenvironment of dystrophic muscle. This shift from mere identification to mechanistic elucidation, particularly for translatable circRNAs, holds potential for uncovering novel therapeutic targets and biomarkers in Duchenne muscular dystrophy. In essence, a complete picture of the disease’s molecular landscape would likely be incomplete without accounting for the potential multifaceted influence of these circular RNAs.

### 4.3 Autoimmune neuromuscular junction disorders (myasthenia gravis)

Myasthenia gravis is a chronic autoimmune disorder characterized by fluctuating muscle weakness and fatigability, caused by autoantibodies targeting components of the neuromuscular junction, most commonly the acetylcholine receptor (AChR^+^), leading to impaired neuromuscular transmission (106). While autoimmunity involving autoantibodies and T-cells is central to myasthenia gravis pathogenesis, recent research has identified significant alterations in circRNA expression and function in myasthenia gravis, highlighting their involvement in immune cell regulation, muscle cell proliferation, and their potential as novel biomarkers and therapeutic targets. These studies reveal a complex landscape where circRNAs act through diverse mechanisms, including miRNA sponging and m^6^A modification influencing mRNA stability.

#### 4.3.1 Dysregulated circRNA expression in myasthenia gravis

Research into circRNA biomarkers in myasthenia gravis has identified several promising candidates. Lv et al. (2021) (25) profiled circRNAs in the peripheral blood of myasthenia gravis patients compared to healthy controls, identifying 162 differentially expressed circRNAs with 87 being upregulated and 75 downregulated. Among these, hsa-circRNA5333-4 was significantly upregulated and validated as a notable biomarker. Its expression demonstrated a strong correlation with the Quantitative Myasthenia Gravis Score and also aligned with gender and acetylcholine receptor antibody levels, underscoring its potential for both myasthenia gravis diagnosis and monitoring disease severity. Similarly, Ye et al. (2023) (107) observed an upregulation of circSRF (hsa_circ_0076490) in the peripheral blood of myasthenia gravis patients compared to healthy controls, further highlighting the biomarker potential of circRNAs in this disease. A recent study by Kong et al., 2024 (108) using microarray analysis on PBMCs identified circFRMD4 (hsa_circ_0004183) and circPIGB (hsa_circ_0035381) as upregulated, while circNUP214 (hsa_circ_0089153) exhibited the lowest expression in AchR^+^ myasthenia gravis patients compared to controls, proposing these as valuable potential novel biomarkers. A study by Lai et al. (109) first established that circFBL (hsa_circ_0051032) was significantly upregulated in both the muscle tissue and the serum of human myasthenia gravis patients compared to healthy controls.

The study by Li et al. (2025) (110) uncovered a novel layer of immune regulation in myasthenia gravis by investigating the epitranscriptomic m^6^A-modified circRNAs in PBMCs from patients and controls. Through bioinformatic analysis, including network construction and integration with existing data, four candidate m^6^A-modified circRNAs (hsa_circ_0084735, hsa_circ_0018652, hsa_circ_0025731, and hsa_circ_0030997) were identified (**Table 2**, **Supplementary L1**).

**Table 2:** Summary of key dysregulated circRNAs in myasthenia gravis (MG).

circRNA (alias)	Participants	Observed dysregulation	Primary proposed mechanism / role in disease context	Ref
hsa-circRNA5333-4	n = 104 MG n = 83 HC	Up-regulated in MG peripheral blood; expression correlates with qMG score & AChR-Ab titres	Predicted ceRNA that sponges miR-4310 and derepresses MORF4L2.	(25)
circSRF (hsa_circ_0076490)	n = 29 MG n = 29 HC	Up-regulated in MG peripheral blood compared to healthy controls	Circ_0076490 silencing inhibits MAPK1 expression to decrease the proliferation and increase apoptosis of Jurkat cells by regulating miR-144-3p.	(107)
circFRMD4 (hsa_circ_0004183)	n = 3 MG n = 3 HC	Up-regulated in AChR^+^-MG PBMCs	Confirmed biding to miR-145-5p, releasing repression on targets such as SMAD4; drives Jurkat cell proliferation and may skew immune balance in MG.	(108)
circNCOA2 (hsa_circ_0084735)	n = 3 MG n = 3 HC	Decreased m^6^A modification level in MG PBMCs	In MG PBMCs, the m^6^A-deficient form was predicted to interact with a regulatory network involvingmiR-183-5p / miR-29c-3p, which in turn impact thetargets genes EGR1, FRAT2, PTGS2.	(110)
circPPFIBP1 (hsa_circ_0025731)	n = 3 MG n = 3 HC	Decreased m^6^A modification in MG PBMCs	Correlates with Th1/Th2-cell balance; predicted miR-29c-3p sponging may modulate cytokine-signalling genes.	(110)
circFBL (hsa_circ_0051032)	n = 35 MG n = 11 HC	Up-regulated in MG muscle / blood	Overexpression of circ‐FBL promoted myoblast proliferation by regulation of miR‐133/PAX7, representing a potential maladaptive regenerative response in MG muscle.	(109)

Most MG circRNA studies to date use PBMCs, exploiting their accessibility for biomarker discovery, but functional validation of several candidates (e.g., circPIGB, circNUP214) is still pending. The m^6^A-epitranscriptomic layer adds an extra dimension: altered methylation status, not just expression level, can remodel the ceRNA capacity of circRNAs and correlate with shifts in specific immune-cell compartments. Muscle-focused circRNAs such as circFBL highlight the possibility that circRNAs simultaneously shape peripheral immunity and the muscle-regeneration milieu that influences neuromuscular junction integrity in MG.

#### 4.3.2 Potential functional roles and mechanistic insights of specific circRNAs in myasthenia gravis

The biomarker candidate hsa-circRNA5333-4 (25) is predicted to interact with hsa-miR-4310, forming a potential competing endogenous RNA network that could involve MORF4L2 (Mortality Factor 4 Like 2). MORF4L2 is known to interact with the tumor suppressor RB, possesses histone deacetylase activity, and participates in cell growth, regulation, and senescence (111). This theoretically suggests that the hsa-circRNA5333-4/hsa-miR-4310/MORF4L2 axis could influence the muscle pathology or adaptive responses in myasthenia gravis, however this remains speculative (25). In Jurkat cells (a human T-cell line), silencing of circSRF, found to be increased in peripheral blood of myasthenia gravis patients, inhibited cell proliferation and promoted apoptosis by modulating the miR-144-3p/MAPK1 (mitogen-activated protein kinase 1) axis (107). Another upregulated circRNA, circFRMD4 (hsa_circ_0004183), has been identified as a modulator of T cell proliferation (108). Upregulated in AchR^+^ myasthenia gravis PBMCs, circFRMD4 has been demonstrated to act as a sponge for miR-145-5p in Jurkat cells (108). By sequestering miR-145-5p, circFRMD4 consequently promotes T-cell proliferation as knockdown of circFRMD4 inhibited proliferation, an effect reversed by inhibiting miR-145-5p. This axis may potentially influence processes via downstream targets of miR-145-5p like SMAD4 involved in T-cell differentiation and Treg regulation (112).

In the muscle and blood of myasthenia gravis patients, significantly increased circFBL has been shown to act as a sponge for miR-133, thereby upregulating PAX7 (Paired Box 7), a key transcription factor for muscle stem cell specification and proliferation (109). Overexpression of circFBL was found to promote myoblast proliferation through this circFBL/miR-133/PAX7 axis in experimental autoimmune myasthenia gravis mice models. Knockdown of circFBL in these mice led to an improvement in clinical symptoms, grip strength and muscle pathology. This suggests that circFBL contributes to myasthenia gravis pathogenesis by influencing myogenic processes, potentially as part of a dysregulated regenerative attempt, which aligns with the hypothesis that impaired myofiber maturation can underlie myasthenia gravis pathology (113).

Epigenetic regulation of circRNA in myasthenia gravis PBMCs revealed (110) four specific m^6^A-modified circRNAs (hsa_circ_0084735, hsa_circ_0018652, hsa_circ_0025731, and hsa_circ_0030997), potentially regulating target genes (including EGR1, FRAT2, PTGS2) by targeting hsa-miR-183-5p and hsa-miR-29c-3p. Crucially, the m^6^A modification levels of these circRNAs correlated with the abundance or activity of different immune cell subpopulations (e.g., m^6^A-hsa_circ_0018652 (circ PPA1) with NK and activated T-cells; m^6^A-hsa_circ_0025731 (circPPFIBP1) with type 2 T-helper cell and type 1 T-helper cell; m^6^A-hsa_circ_0030997 (circLAMP1) with macrophages, memory CD4 T-cells, and dendritic cells). Furthermore, the m^6^A-modified levels of circNCOA2 (hsa_circ_0084735) and circPPFIBP1 (hsa_circ_0025731) were decreased in myasthenia gravis patients. These findings suggest that m^6^A modification of specific circRNAs plays a role in regulating immune cell populations and their functions in myasthenia gravis and altered m^6^A levels might disrupt these regulatory networks.

The growing body of evidence firmly establishes circRNAs as significant, multi-faceted players in the intricate pathology of myasthenia gravis. From their emergence as promising diagnostic and severity biomarkers in peripheral blood to their predicted involvement in immune cell regulation and muscle regenerative processes, circRNAs are clearly integral to myasthenia gravis’ complex landscape. While these initial findings provide valuable insight, primarily from PBMCs, a deeper understanding of circRNA functions directly within neuromuscular junction and muscle tissues is still largely lacking. Rigorous validation in larger clinical cohorts and in-depth functional studies are now essential to fully delineate their precise contributions to myasthenia gravis pathogenesis.

## 5 Future perspectives and conclusion

### 5.1 Engineered circular RNA

Recent pioneering studies demonstrate the significant therapeutic potential of engineered circRNAs for immune-related muscle diseases. The first study, by creating an artificial circRNA sponge (circmiR-29b) that potently inhibits the pro-atrophic microRNA miR-29b, provides a powerful proof-of-concept for treating muscle wasting, a common feature in many myopathies, such as myopathy driven by TNF-α (114). The second study moves beyond sponges, engineering an antisense circular RNA capable of highly efficient exon skipping to restore dystrophin expression in Duchenne muscular dystrophy. Together, these approaches highlight how circRNA technology can be harnessed not only to neutralize detrimental pathways like atrophy but also to correct underlying genetic defects, offering versatile and potentially more stable therapeutic strategies for a range of devastating neuromuscular disorders (115).

### 5.2 Translational challenges for engineered circRNA therapeutics

The clinical translation of engineered circRNAs for muscular immune-related diseases faces several critical hurdles, primarily revolving around delivery, safety, and manufacturing. Despite their therapeutic promise stemming from high stability and prolonged expression, realizing their potential requires overcoming the following challenges:

- Efficient delivery to target tissues: As large, anionic macromolecules, circRNAs cannot passively cross cell membranes. Their delivery to skeletal muscle and immune cells is a primary barrier, often addressed with nano-delivery platforms (116–118).
  -Viral Vectors: Adeno-associated viruses (AAVs) show promise for muscle delivery but are constrained by limited packaging capacity (~4.7 kb) and potential immunogenicity from pre-existing neutralizing antibodies.
  -Lipid Nanoparticles (LNPs): LNPs are a leading non-viral platform, utilizing ionizable lipids for endosomal escape. However, achieving robust extrahepatic delivery remains a major challenge, though novel selective-organ-targeting chemistries are expanding their reach to immune cell subsets like B and T cells.
  -Extracellular Vesicles (EVs): EV-based carriers offer low immunogenicity and natural tropism, but face significant obstacles in scalable manufacturing and consistent cargo loading.
- Achieving cell-type precision in inflamed muscle: In diseased tissue, infiltrating immune cells and myofibers are in close proximity, creating a high risk of off-target delivery. Achieving cellular precision is therefore paramount. Strategies to achieve cellular precision include (116):
  -Active Targeting: Decorating delivery vectors with ligands (e.g., antibodies against T-cell markers) to direct them to specific cell populations.
  -Genetic Targeting: Using cell-type-specific promoters (e.g., muscle creatine kinase promoter) within AAV cassettes to restrict circRNA expression to myofibers.
- Mitigating innate immune sensing: While circularization inherently reduces sensing by 5’/3’ end-recognizing receptors like RIG-I, impurities in synthetic preparations can trigger inflammatory responses (119).
  -Contaminants: Linear and double-stranded RNA (dsRNA) by-products are potent activators of RIG-I, MDA5, and TLR pathways.
  -Mitigation: Rigorous purification using methods like High-Performance Liquid Chromatography is essential. Careful sequence design and the potential incorporation of modified nucleotides can further minimize immunogenicity.
- Scalable manufacturing and quality control: Clinical translation requires reproducible, large-scale production of high-purity circRNAs. While enzymatic and rolling-circle amplification methods are improving yields, robust analytical assays are needed to quantify circularization efficiency, purity, and integrity as key quality attributes (120).
- Controlling expression and off-target effects: The exceptional stability of circRNAs is a double-edged sword, making it difficult to reverse their effects. For protein-coding circRNAs, adding cell-restricted elements or miRNA response elements can limit expression in non-intended cells. For antisense and sponge designs, comprehensive in silico prediction and transcriptome-wide off-target profiling are needed before *in vivo* studies (121).

In summary, advancing circRNA therapies for muscle-immune diseases hinges on developing tailored, high-precision delivery systems, establishing stringent manufacturing and purification standards, and designing robust safety controls to manage their potent and long-lasting biological activity.

### 5.3 Research directions

Future research into circRNAs in muscle–immune diseases presents several critical avenues to advance our understanding and therapeutic capabilities.

First, comprehensive and well-powered circRNA profiling in diverse patient cohorts and relevant tissues is paramount. While initial studies have provided valuable insights into dysregulated circRNAs in specific contexts (e.g., muscle biopsies for idiopathic inflammatory myopathies subtypes, myoblast cultures and mdx mouse models for Duchenne muscular dystrophy, PBMCs and muscle tissue for myasthenia gravis), a broader and more granular understanding is needed. This includes profiling not only affected muscle tissue but also sorted immune cell populations (T-cells, B-cells, macrophages) and readily accessible biofluids (serum/plasma). Such comprehensive data will be crucial for identifying disease-specific and subtype-specific circRNA signatures and accessible biomarkers for diagnosis, prognosis, and monitoring treatment response (1, 4).

Second, rigorous functional validation of dysregulated circRNAs is a critical next step. Elucidating their precise molecular mechanisms, such as their capacity as miRNA sponges, their interactions with proteins, their potential for cap-independent translation into functional proteins, or the influence of m^6^A modification on their stability and function, is needed. These investigations should extend beyond *in vitro* models to appropriate *in vivo* animal models that accurately recapitulate disease pathology. Understanding how these circRNAs impact key disease pathways, including inflammation, muscle differentiation and regeneration, immune cell function (e.g., polarization and activation), and muscle wasting, will be vital for determining their pathogenic roles. For instance, further investigation into circHIPK3 which was observed to be altered across multiple disease-relevant cell types and models (myoblast proliferation, macrophage inflammation, mdx muscles), could reveal a central, conserved role, or distinct context-dependent functions. Similarly, CDR1as merits further study, given its roles in immune cells and satellite cells, even though it hasn’t been directly implicated in these three specific diseases yet.

Third, longitudinal studies in larger, diverse patient cohorts are indispensable. Such studies are essential to validate the diagnostic, prognostic, and treatment-response biomarker potential of candidate circRNAs. While some promising biomarker candidates have been identified (e.g., hsa-circRNA5333-4 in myasthenia gravis peripheral blood correlating with disease severity), independent validation in larger cohorts is needed to confirm their clinical utility.

A notable gap in current research is the simultaneous identification and validation of circRNAs in both affected tissues and accessible blood samples within the same studies, which is crucial for developing clinically relevant biomarkers. Until now, analyses also did not address disease subtypes heterogeneity within samples analyzed for circRNA profiles.

Finally, the burgeoning field of engineered circRNAs holds substantial therapeutic promise. Building on pioneering studies that demonstrate the ability to create artificial circRNA sponges or antisense circRNAs to modulate detrimental miRNAs or restore protein expression. While circRNAs remain highly promising as next-generation diagnostics and therapeutics, successful translation will depend on solving delivery, specificity, and innate-immunity challenges, alongside rigorous *in vivo* validation in disease-relevant muscle and immune cell contexts. Besides, assessing long-term efficacy and safety is crucial.

## 6 Conclusion

CircRNAs are emerging as versatile regulators across muscle–immune diseases, shaping myogenesis and regeneration, interfacing with innate and adaptive immunity, and offering a potential biomarker and therapeutic potential. However, most mechanistic depth still derives from non-human systems or peripheral blood rather than human skeletal muscle or neuromuscular junctions; accordingly, current findings should be treated as hypothesis-generating for humans. Comparative analyses indicate that only a minority of circRNAs show conserved splice-site usage between human and mouse (≈5–30%), underscoring isoform-level divergence that complicates extrapolation (122). Species-specific context—differences in fiber composition, immune infiltration, cytokine milieu and regenerative capacity as well as differences in presence of target miRNA—can further shift circRNA biogenesis, localization, and target engagement, weakening one-to-one mapping from animal models to human disease. We therefore conclude that existing animal findings motivate rather than establish circRNA mechanisms in human skeletal muscle and myopathies.

In idiopathic inflammatory myopathies, circRNA datasets remain early and underpowered. In Duchenne muscular dystrophy, altered circRNA landscapes in patient myoblasts and mdx muscle align with impaired differentiation, inflammation, and calcium/cAMP signaling, and selected circRNAs show coding potential, but human in-tissue causality is not yet demonstrated. In myasthenia gravis, peripheral circRNA biomarkers are promising and initial functional leads (e.g., T-cell programs, MuSC regulators) are encouraging, yet direct evidence within patient muscle and specifically neuromuscular junction.

Overall, circRNAs likely contribute to the pathophysiology of muscle–immune diseases, but definitive human mechanisms await for further functional (isoform-accurate back splice junction detection in human muscle and relevant immune and back splice junction-specific loss-and-rescue experiments in appropriate models) and clinical association and replication. Until such evidence accrues, circRNAs are best positioned as plausible contributors and promising biomarker/therapy candidates, not yet as established drivers of human muscle–immune disease.
