# Supplementary material for: Circular RNAs and their emerging roles in muscular immune-related diseases
Source: Front Immunol. 2025 Nov 13;16:1675567. doi: 10.3389/fimmu.2025.1675567 (PMC12657405; doi:10.3389/fimmu.2025.1675567)
Supplement: Supplementary file 1 [file DataSheet1.docx]

Circular RNAs and Their Emerging Roles in Muscular Immune-Related Diseases

Supplementary Material

# Supplementary List L1

**List of circRNA corresponding to manuscript chapters**

*3.1 Dynamic Regulation of Myogenesis, Muscle Stem Cell Function, and Regeneration by circRNAs in Muscle:*

1. circINSR (bovine): circRNA regulates cell proliferation and apoptosis by sponging miR-34a, which in turn modulates Bcl-2 and CyclinE2 expression. It is typically more active during early developmental stages and in embryonic muscle tissues and is associated with decreased apoptosis (51).

2. circLMO7 (bovine): Sponges miR-378a-3p, thereby upregulating HDAC4 to promote myoblast proliferation and inhibit differentiation. Overexpression of HDACs like HDAC4/5 inhibits myogenic differentiation and prevents premature activation of muscle-specific gene programs (54).

3. circHUWE1 (bovine): Enhances AKT signaling and promotes cell proliferation. It acts as a sponge for miR-29b, preventing this miRNA from suppressing its target, AKT3, leading to increased AKT3 expression and activity (57).

4. circRBFOX2 (chicken): Enhances myoblast proliferation by acting on the miR-206/CyclinD2 axis (52). This promotes the expansion of muscle precursor cells.

5. circFGFR4 (bovine): circRNA promotes myoblast differentiation by binding miR-107, which relieves miR-107's inhibition of Wnt3a/β-catenin. This action induces the expression of MyHC, MyoD, and MyoG (59).

6. circSNX29 (bovine): Promotes myoblast differentiation by sponging miR-744, thereby activating the Wnt5a/Ca²⁺ signaling pathway via CaMKIIδ/NFATC1 activation of myogenic genes (60).

7. circMEF2A1 (chicken and mouse): circRNA promotes differentiation by regulating the miR-30a-3p/PPP3CA/NFATC1 axis (61).

8. circMEF2A2 (chicken and mouse): Promotes differentiation by targeting the miR-148a-5p/SLIT3/ROBO2/β-catenin signaling pathway (61).

9. circTGFβ2 (goats): It promotes myoblast differentiation by sponging miR-206 and miR-211, which alleviates their suppression of crucial myogenic markers like MyoD, MyoG, and MyHC (62).

10. circMYBPC1 (cattle): Promotes cell differentiation by directly binding to the Myosin Heavy Chain (MyHC) protein, increasing MyHC expression at both mRNA and protein levels. It also promotes myoblast differentiation by directly binding miR-23a, relieving the miRNA's inhibitory effect on MyHC expression (63).

11. circSamd4 (mouse C2C12 myoblasts): Overexpressed during skeletal muscle differentiation, it enhances MyHC production by reducing the binding of PURA and PURB proteins (which normally repress MyHC) to the MHC promoter (43).

12. circKANSL1L (porcine): It is translated into a functional protein. This circKANSL1L-derived protein directly interacts with Akt, promoting the phosphorylation of FoxO3 and ultimately activating the Akt-FoxO signaling pathway to drive differentiation (64).

13. circSVIL (chicken): Promotes both myoblast proliferation and differentiation by targeting miR-203, thereby regulating MEF2C enhancer factor (65).

14. CircHIPK3 (chicken, mouse, human): Exhibits dual functionality: in chicken myoblasts sponges miR-30a-3p to upregulate MEF2C (66), and in mouse C2C12 cells, its overexpression reversed the inhibitory effect of miR-7 on both proliferation and differentiation by increasing Transcription Factor 12 (TCF12) expression (67). In macrophages, it acts as a pro-inflammatory circRNA by sponging miR-192 and miR-561, leading to significantly upregulated NLRP3 expression and enhancing the assembly and activation of the NLRP3 inflammasome (97).

15. circTTN (bovine): It enhances both proliferation and differentiation phases by sponging miR-432, which increases the protein expression of Insulin-like Growth Factor-2 (IGF2) and key components of the PI3K/AKT signaling pathway, such as IRS1, PI3K, PDK1, and AKT (69).

16. circUSP13 (goats): Activates essential signaling cascades like the PI3K/AKT pathway by sequestering miR-29c, which targets Insulin-like Growth Factor 1 (IGF1) (70).

17. CDR1as (ciRS-7) (goats, mouse): Enhances goat muscle satellite cell (MuSC) differentiation by acting as a sponge for miR-7, leading to increased levels of its target, IGF1R, a key promoter of differentiation. Its transcription is activated by MyoD, forming a positive feedback loop that reinforces the myogenic program (58). In mice macrophages, it acts as an anti-inflammatory brake, skewing macrophages toward an M2 phenotype and suppressing M1 markers (96).

18. circPAPD7 (goats): In goat MuSCs, it sponges miR-26a-5p, relieving the suppression of EZH2, a crucial epigenetic regulator for maintaining stem cell self-renewal and proliferation by partly repressing differentiation-specific genes (55).

19. circFAM188B (chicken): It is translated into peptide circFAM188B-103aa which modulates the cell cycle, leading to increased proliferation and inhibited differentiation (56).

20. CircFUT10 (human): Notably upregulated in aged MuSCs, it inhibits proliferation and differentiation. Mechanistically, circFUT10 acts as a sponge for miR-365a-3p. By competitively binding this miRNA, circFUT10 attenuates the normal suppressive effect of miR-365a-3p on its target, HOXA9 (123).

21. circFNDC3AL (chicken): It enhances both MuSC proliferation and differentiation by sponging miR-204, which upregulates BCL9, a critical co-activator for Wnt signaling and a regulator of differentiation-related genes (68).

22. CircPPP1R13B (chicken): Facilitates MuSC proliferation and differentiation by sponging miR-9-5p. This de-represses IGF2BP3 expression, consequently triggering the IGF/PI3K/AKT signaling pathway, which is vital for both cell growth and myogenic progression (71).

23. circFgfr2 (mouse): In mouse models of CTX skeletal muscle injury, it promotes muscle regeneration primarily by sponging miR-133. This sequestration alleviates the suppression of miR-133's target, Map3k20, leading to the activation of the JNK/MAPK signaling pathway. This pathway activation is associated with a feedback loop involving the transcription factor Klf4, contributing to cell differentiation and regeneration (21).

24. circRILPL1 (mouse): In mouse models of skeletal muscle injury it promotes muscle regeneration by targeting miR-145, which influences Insulin-like Growth Factor-1 Receptor (IGF1R) levels. This subsequently activates the PI3K/AKT pathway, inducing myoblast proliferation and differentiation (72).

25. circAGGF1 (mouse): In mouse models skeletal muscle injury induced by CTX regulates myogenic processes by acting as a sponge for miR-199a-3p. This prevents miR-199a-3p from targeting and suppressing Fibroblast Growth Factor 7 (Fgf7), leading to increased Fgf7 levels and upregulation of canonical myogenic markers (73).

26. circCPE (mouse): In mouse models of skeletal muscle injury induced by CTX counteracts the inhibitory effect of miR-138 on cell proliferation and the enhancing effect on apoptosis and differentiation. This combined effect, forcing proliferation while blocking differentiation, ultimately leads to defective muscle regeneration (74).

*3.2. Muscle wasting:*

27. CircAGO3 (chicken): Derived from the AGO3 gene, it is highly expressed in atrophying chicken muscle and acts as a sponge for miR-34b-5p, relieving inhibition on TRAF3, an upstream activator of NF-κB signaling. Enhanced NF-κB activity increases expression of atrophy markers, linking circAGO3 to inflammation-driven muscle atrophy (15).

28. circTmeff1 (mouse): Promotes muscle loss by indirectly activating the NF-κB pathway. It binds the RNA-binding protein TDP-43 and sequesters it in mitochondria, triggering the release of mitochondrial DNA and activation of the cGAS-STING innate immune pathway. This leads to NF-κB and interferon signaling that exacerbates muscle protein breakdown. Knockdown of circTmeff1 blunts NF-κB–associated gene induction and partially rescues muscle mass in diverse atrophy models. Its involvement was also confirmed in various in vitro models of muscle atrophy induced by dexamethasone treatment, TNF-α, or Ang II treatment (76).

29. circSmox (mouse): Upregulated in models of glucocorticoid-induced atrophy, such as C2C12 myotubes treated with dexamethasone. Its upregulation is accompanied by co-elevated levels of p21 mRNA, consistent with a decrease in cell proliferation, a characteristic feature observed in glucocorticoid-induced muscle atrophy (89). Another study shows that CircSmox knockdown alleviates PC12 cell apoptosis and inflammation in spinal cord injury by miR-340-5p/Smurf1 axis (124).

30. circDdb1 (mouse): Upregulated across multiple atrophy models. Overexpression of circDdb1 is sufficient to cause muscle fiber atrophy, whereas silencing it mitigates muscle wasting induced by Ang II, TNF-α, or dexamethasone treatment. It is produced from the DDB1 gene and encodes a novel 867-amino acid protein (circDdb1-867aa). This protein binds to eukaryotic elongation factor 2 (eEF2) and enhances inhibitory eEF2 phosphorylation at Thr56, which decreases protein translation and contributes to muscle mass loss (91).

31. circCCDC91 (chicken): Identified as downregulated under dexamethasone treatment in a chicken muscle. Its overexpression significantly alleviated the atrophic effects induced by dexamethasone. Mechanistically, circCCDC91 sponges the miR-15/16 family, leading to the upregulation of Insulin Receptor Substrate 1 (IRS1), which reactivates IGF-1–PI3K–Akt signaling, thereby attenuating muscle atrophy (90).

32. circTMTC1 (chicken): Abundantly expressed in atrophic chicken muscle, this pro-atrophy circRNA contributes to muscle loss by inhibiting myoblast differentiation through its action as a sponge for miR-128-3p. By sequestering miR-128-3p, circTMTC1 effectively increases myostatin levels, a key negative regulator of muscle mass, leading to reduced muscle development and mass loss (18).

33. circANAPC7 (human): Recently characterized in a cancer cachexia model, it sponges miR-373, which is induced by cachectic factors, preventing miR-373 from downregulating PHLPP2, a phosphatase that activates AKT. The restoration of AKT activity by circANAPC7 leads to a reduction in muscle proteolysis. Additionally, circANAPC7's action dephosphorylates and inactivates STAT5, which in turn reduces the secretion of TGF-β from muscle (81).

*3.3 circRNAs in Immune Cells Driving Inflammation:*

34. circIKZF1 (human): Found to be overexpressed in T-cells (24).

35. circTNIK (human): Found to be overexpressed in T-cells (24).

36. circTXK (human): Found to be overexpressed in T-cells (24).

37. circFBXW7 (human): Found to be overexpressed in T-cells (24).

38. circPAX5 (human): Originating from a key B-lineage transcription factor, it is essential for controlling B-lymphocyte differentiation and preserving B-cell identity, potentially by influencing gene expression patterns unique to B-cell maturation (24).

39. CircAFF3 (human): Displays distinct expression patterns in B-cells, assisting in the regulation of their differentiation and activity (24).

40. CircIL4R (human): Displays distinct expression patterns in B-cells, assisting in the regulation of their differentiation and activity. It may also contribute to B-cell activation and enhance the immune response in conjunction with interleukin receptor signaling (24).

41. CircSETBP1 (human): Displays distinct expression patterns in B-cells, assisting in the regulation of their differentiation and activity (24).

42. CircNUP214 (human): Upregulated in the CD4+ T cells of rheumatoid arthritis patients and has been shown to promote Th17 differentiation (94).

43. CircINPP4B (mice): Enhances Th17 cell differentiation by sequestering miR-30a, suggesting it as a potential therapeutic target in autoimmune diseases (95).

44. CircCdr1as (mice): Loos of circCdr1as in muscle-infiltrating macrophages could exacerbate inflammation (96).

45. CircHIPK3 (synovial tissue mononuclear cells of patients with gouty arthritis): Overexpression in macrophage exemplifies pro-inflammatory role, by sponging miR-192 and miR561, thereby activate the NLRP3 inflammasome (97).

46. CircPPM1F (PBMC of T1DM patients): Identified as a positive regulator of classically activated (M1) macrophages via stabilization of NF-κB signaling components (98).

47. circRNF19B (hsa_circ_0000048) (human THP1 cell line): Significantly more highly expressed in the M1 macrophage phenotype and actively promotes macrophage polarization towards pro-inflammatory M1, partly through miRNA sponging (e.g., of miR-217, miR-6274-5p) (23).

48. circRasGEF1B (mcircRasGEF1B) (mouse RAW264.7 and human THP1 cell line): A novel, LPS-inducible cytoplasmic circRNA, conserved between humans and mice (99).

*4.1* *Idiopathic inflammatory myopathies (IIMs):*

*While the dysregulation of circRNAs in inflammatory myopathy samples was observed by Tsitsipatis et al. (2022) (103), their functional significance in idiopathic inflammatory myopathies (IIMs) remains largely unexplored. The following discussion presents hypotheses regarding potential mechanisms, drawing upon reported roles of these dysregulated circRNAs in other biological systems. However, this remains a speculative model that requires direct experimental testing in IIM-specific cellular models (e.g., patient-derived myoblasts or immune cells) and animal models of IIMs to validate both its functional impact and mechanistic relevance.*

1. Observation in Myopathy Samples: circITGB6 (hsa_circ_0056856): Downregulated in human myopathy samples, including inflammatory myopathies, relative to healthy controls (103);

Reported mechanism(s) in other systems and hypotheses derived from these observations: Drawing from cancer research, the TGFβ-inducible circITGB6 has been shown to form a circITGB6/IGF2BP3/PDPN axis, which upregulates Podoplanin (PDPN). Given that PDPN is a known driver of M1 macrophage polarization, it is hypothesized that the observed dysregulation of circITGB6 in myopathy samples, specifically its downregulation, could potentially disrupt this axis and contribute to an altered inflammatory microenvironment in muscle diseases. This suggests circITGB6 as a possible candidate for further investigation in IIMs (125).

2. Observation in Myopathy Samples: circZCCHC2 (hsa_circ_0047886): Downregulated in human myopathy samples, including inflammatory myopathies, relative to healthy controls (103);

Reported mechanism(s) in other systems and hypotheses derived from these observations: In other disease contexts, such as hepatocellular carcinoma (HCC), circZCCHC2 has identified as an oncogenic factor that modulates the miR-936/BTBD7/Rho/ROCK2 pathway. The broader significance of this pathway is noteworthy, as its downstream effector, ROCK2 (Rho-associated coiled-coil containing protein kinase 2), is a critical regulator of both immune modulation and fibrosis. Therefore, it is hypothesized that the observed downregulation of circZCCHC2 in myopathy samples, or dysregulation of related mechanisms, could lead to altered ROCK2 activity. This altered activity, particularly an upregulation of ROCK2, is plausibly implicated in the pathology of immune-related muscle diseases by potentially contributing to chronic inflammation and the development of fibrotic tissue within muscle. These findings implicate circZCCHC2 as a candidate for further investigation in IIMs (126).

3. Observation in Myopathy Samples: circALPK2 (hsa_circ_0141401): Downregulated in human myopathy samples, including inflammatory myopathies, relative to healthy controls (103);

Reported mechanism(s) in other systems and hypotheses derived from these observations: In cardiac models, circALPK2 has been identified as a negative regulator of cell proliferation by modulating the miR-9/GSK3B axis inhibiting the Wnt/β-catenin pathway. Given that the Wnt signaling pathway plays conserved, critical roles in skeletal muscle regeneration and immune cell modulation—two processes central to IIM pathogenesis—circALPK2 emerges as an intriguing candidate for further investigation in IIMs. Its downregulation in inflammatory myopathy samples hints at a potential role in disrupting Wnt-mediated muscle-immune crosstalk; however, this remains a speculative model that requires direct experimental testing in IIM cellular and animal models (127).

4. Observation in Myopathy Samples: circNPHP1 (hsa_circ_0117010): Downregulated in human myopathy samples, including inflammatory myopathies, relative to healthy controls (103);

Reported mechanism(s) in other systems and hypotheses derived from these observations: In human heart, the circNPHP1/miR-221-3p/VEGFA axis has been identified as a crucial pro-angiogenic pathway, that promotes new blood vessel formation. Human endothelial cells were used to show that circNPHP1 binds miR-221-3p and with this increases VEGFA and BCL2. Considering that microvascular ischemia and impaired angiogenesis are known contributors to muscle fiber necrosis in conditions like dermatomyositis, it is hypothesized that the observed downregulation of circNPHP1 in myopathy samples could lead to a disruption of this pro-angiogenic pathway. This disruption might result in impaired blood vessel formation, which is plausibly implicated in the pathology of immune-related muscle diseases by contributing to muscle fiber damage and necrosis (128).

5. Observation in Myopathy Samples: circCCDC9 (hsa_circ_0000944): Downregulated in human myopathy samples, including inflammatory myopathies, relative to healthy controls (103);

Reported mechanism(s) in other systems and hypotheses derived from these observations: The circular RNA circCCDC9 has been shown to modulate the expression of Caveolin-1 (CAV1) by acting as a sponge for miR-6792-3p in gastric cancer. As CAV1 is essential for muscle membrane integrity and repair, and is also plays a role in fibrotic processes, it is hypothesized that the observed downregulation of circCCDC9 in myopathy samples could lead to altered CAV1 expression. This altered regulation of the circCCDC9/miR-6792-3p/CAV1 pathway needs to be tested and validated in immune-related muscle diseases to position this circRNA as a potential contributor to the myofiber damage and fibrosis characteristic of these chronic disorders (129).

6. Observation in Myopathy Samples: circZNF362 (hsa_circ_0009027): Downregulated in human myopathy samples, including inflammatory myopathies, relative to healthy controls (103); a zinc finger regulator of transcription, specific function is unknown.

7. Observation in Myopathy Samples: circAMY2B (hsa_circ_0000099): Upregulated in human myopathy samples, including inflammatory myopathies, relative to healthy controls (103);

Reported mechanism(s) in other systems and hypotheses derived from these observations: The TGF-β2/circ_0000099/miR-223-3p/CTGF axis identified in study of human lens epithelial cells presents a molecular link between the inflammatory signal TGF-β and the upregulation of the potent pro-fibrotic factor, CTGF. Therefore, it is hypothesized that the observed upregulation of circAMY2B in myopathy samples could be involved in the activation or regulation of the fibrotic pathway. This model, however, remains speculative and requires direct experimental validation in relevant IIM models (130).

8. Observation in Myopathy Samples: circARHGAP12 (hsa_circ_0000231): Upregulated in human myopathy samples, including inflammatory myopathies, relative to healthy controls (103);

Reported mechanism(s) in other systems and hypotheses derived from these observations: Studies in other systems highlight the versatile roles of circARHGAP12. It has been shown to potentially encode a protective protein, act as a non-coding miRNA sponge to regulate autophagy, or be m^6^A-modified to control cellular metabolism via a major oncogene. This demonstrates that its biological role is highly context-dependent, and it can influence fundamental cellular processes such as cell survival, autophagy, and metabolism through diverse molecular mechanisms. Given this versatility, it is hypothesized that the observed upregulation of circARHGAP12 in myopathy samples could, depending on the specific cellular context of immune-related muscle diseases, contribute to altered cell survival, dysregulated autophagy, or metabolic reprogramming within muscle or immune cells. Further investigation is needed to determine which of these diverse mechanisms, or others, might be relevant in muscle pathologies (131–133).

*4.2 Duchenne muscular dystrophy (DMD):*

1. circ-ZNF609 (human and mouse): Persistently upregulated in DMD myoblasts, impairs differentiation and promotes proliferation; encodes a protein (53).

2. circ-QKI (human and mouse): Downregulated in DMD myoblasts, its loss correlates with delayed myogenic differentiation (53).

3. circ-BNC2 (human and mouse): Downregulated in DMD myoblasts, its reduction is linked to impaired differentiation (53).

4. circHIPK3 (mmu_circRNA_19008) (mouse): Upregulated in mdx mouse model muscle (92), may disturb miRNA networks (e.g., miR-186-5p) supporting muscle regeneration (105).

5. circMpdz (mmu_circRNA_36990) (mouse): Downregulated in mdx mouse model muscle; predicted to encode a novel peptide (104).

6. circIde (mmu_circRNA_32522) (mouse): Downregulated in mdx mouse model muscle; a predicted protein-coding circRNA (104).

7. circErc1 (mmu_circRNA_40856) (mouse): Downregulated in mdx mouse model muscle; predicted to translate (104).

8. circZfp423 (mmu_circRNA_43272) (mouse): Upregulated in mdx mouse model muscle; predicted protein-coding potential, Zfp423 is linked to muscle/adipogenic lineage (104).

9. circPlcl2 (mmu_circRNA_19191) (mouse): Upregulated in mdx mouse model muscle; predicted protein-coding potential, may participate in calcium/phospholipid signaling (104).

*4.3 Myasthenia gravis (MG):*

1. hsa-circRNA5333-4 (human): Upregulated in MG peripheral blood, correlates with qMG score and AChR-Ab titres (25); predicted to sponge miR-4310, affecting MORF4L2 (111).

2. circSRF (hsa_circ_0076490) (human): Upregulated in MG peripheral blood; its silencing inhibits T-cell proliferation and promotes apoptosis by modulating the miR-144-3p/MAPK1 axis (107).

3. circFRMD4 (hsa_circ_0004183) (human): Upregulated in AChR+ MG PBMCs (107); sponges miR-145-5p to promote T-cell proliferation, potentially via SMAD4 (112).

4. circPIGB (hsa_circ_0035381) (human): Upregulated in AChR+ MG PBMCs; proposed as a biomarker, mechanism unknown (108).

5. circNUP214 (hsa_circ_0089153) (human): Downregulated in AChR+ MG PBMCs; proposed as a biomarker, mechanism unknown (108).

6. circNCOA2 (hsa_circ_0084735) (human): A regulatory network was constructed in MG PBMCs, identifying miR-183-5p and miR-29c-3p as regulators of the target genes EGR1, FRAT2, and PTGS2. Based on this network, the m^6^A-deficient form was predicted to alter the activity of these miRNAs. (110).

7. circPPA1 (hsa_circ_0018652) (human): Experimentally validated, the m^6^A methylation level of hsa_circ_0018652 was inversely correlated with the proportion of CD56dim natural killer cells but positively correlated with the proportions of total natural killer and activated T cells. However, the overall m^6^A level of this circRNA did not differ significantly between the myasthenia gravis and control groups. (110).

8. circPPFIBP1 (hsa_circ_0025731) (human): It was identified as a dysregulated circRNA in myasthenia gravis. The m^6^A-modified level of hsa_circ_0025731 has significantly positive correlations with the type 2 T-helper cell and type 1 T-helper cell (110).

9. circLAMP1 (hsa_circ_0030997) (human): Altered m^6^A levels in MG PBMCs (not significantly different overall) link to macrophage, dendritic cell, and memory CD4+ T-cell populations (110).

10. circFBL (hsa_circ_0051032) (human, validation in mouse): Upregulated in MG muscle/blood; sponges miR-133 to elevate PAX7, promoting myoblast proliferation (109,113).

**Additional reference for the Supplementary List L1**

123. Zhu M, Lian C, Chen G, Zou P, Qin BG. CircRNA FUT10 regulates the regenerative potential of aged skeletal muscle stem cells by targeting HOXA9. Aging (Albany NY) (2021) 13:17428–17441. doi: 10.18632/aging.203233

124. Han Z, Mou Z, Jing Y, Jiang R, Sun T. CircSmox knockdown alleviates PC12 cell apoptosis and inflammation in spinal cord injury by miR‐340‐5p/Smurf1 axis. Immun Inflamm Dis (2023) 11:e824. doi: 10.1002/iid3.824

125. Li K, Guo J, Ming Y, Chen S, Zhang T, Ma H, Fu X, Wang J, Liu W, Peng Y. A circular RNA activated by TGFβ promotes tumor metastasis through enhancing IGF2BP3-mediated PDPN mRNA stability. Nat Commun (2023) 14:6876. doi: 10.1038/s41467-023-42571-1

126. Yin J, Wang M, Chen J, Li H, Zhuo J, Lu B, Cai Y. CircZCCHC2 (hsa_circ_0000854) promotes hepatocellular carcinoma progression through modulating miR-936/BTBD7 axis and activating Rho/ROCK2 pathway. Noncoding RNA Res (2024) 9:437–446. doi: 10.1016/j.ncrna.2023.12.004

127. Wu H, Jiang X, Fan H, Li J, Li Y, Lin Y, Zhao D, Han X, Yu M, Tang J-M, et al. Inhibition of circALPK2 enhances proliferation and therapeutic potential of human pluripotent stem cell-derived cardiomyocytes in myocardial infarction. Stem Cell Res Ther (2025) 16:107. doi: 10.1186/s13287-025-04230-8

128. Anwar M, Sarkar M, Ford K, Angelini GD, Punjabi P, Laftah A, Chamorro-Jorganes A, Ji J, Srivastava PK, Petretto E, et al. circRNA-miRNA-mRNA networks reveal a proangiogenic action of circNPHP1 in human ischemic heart disease. (2024)2024.06.04.597402. doi: 10.1101/2024.06.04.597402

129. Luo Z, Rong Z, Zhang J, Zhu Z, Yu Z, Li T, Fu Z, Qiu Z, Huang C. Circular RNA circCCDC9 acts as a miR-6792-3p sponge to suppress the progression of gastric cancer through regulating CAV1 expression. Mol Cancer (2020) 19:86. doi: 10.1186/s12943-020-01203-8

130. Tang H, Shu S, Hu S, Chen L. Circ_0000099/miR-223-3p/CTGF Regulates the Growth, Metastasis, and EMT Processes in TGF-β2-Stimulated Human Lens Epithelial Cells. Curr Eye Res (2024) 49:1042–1053. doi: 10.1080/02713683.2024.2357600

131. Liu D, Zhao X, Wang Z, Wang G, Chen Z, Ning S, Feng D, Sun X, Sun R, Yao J, et al. A novel protein encoded by circARHGAP12 attenuates DNA damage and apoptosis by regulating MDC1 in intestinal ischemia/reperfusion injury. International Journal of Biological Macromolecules (2025) 286:138374. doi: 10.1016/j.ijbiomac.2024.138374

132. Meng F, Shen F, Ling H, Jin P, Zhou D, Li Q. CircARHGAP12 Triggers Mesenchymal Stromal Cell Autophagy to Facilitate its Effect on Repairing Diabetic Wounds by Sponging miR-301b-3p/ATG16L1 and miR-301b-3p/ULK2. J Invest Dermatol (2022) 142:1976-1989.e4. doi: 10.1016/j.jid.2021.11.039

133. Zhang D, Guo Q, You K, Zhang Y, Zheng Y, Wei T. m6A-modified circARHGAP12 promotes the aerobic glycolysis of doxorubicin-resistance osteosarcoma by targeting c-Myc. J Orthop Surg Res (2024) 19:33. doi: 10.1186/s13018-023-04502-0
